# Trimeric Class I Viral Fusion Protein Vaccine Immunogens Using a Trimeric Autotransporter in a Killed Whole-Cell Bacteria Vaccine Platform: Applications to HIV MPER

**DOI:** 10.3390/v18080890

**Published:** 2026-08-13

**Authors:** Juan Sebastian Quintero-Barbosa, Yufeng Song, Frances Mehl, Shubham Mathur, Lauren Livingston, Xiaoying Shen, David C. Montefiori, Steven L. Zeichner

**Affiliations:** 1 Department of Pediatrics, University of Virginia, Charlottesville, VA 22908, USA; pye9my@virginia.edu (J.S.Q.-B.); bxw6yz@virginia.edu (Y.S.); mathur.shubham1990@gmail.com (S.M.); fja9zg@virginia.edu (L.L.); 2 Department of Surgery, Duke University, Durham, NC 27710, USA; sxshen@duke.edu (X.S.); monte@duke.edu (D.C.M.); 3 Duke Human Vaccine Institute, Duke University, Durham, NC 27710, USA; 4 Department of Microbiology, Immunology, and Cancer Biology, University of Virginia, Charlottesville, VA 22908, USA

**Keywords:** HIV-1, MPER, gp41, trimeric antigen presentation, killed whole-cell vaccine, genome-reduced bacteria, Hia autotransporter, rational vaccine design, neutralizing antibodies, broadly neutralizing antibodies, class I viral fusion protein stalk vaccine, immunomodulator engineering

## Abstract

Background: Trimeric envelope-proximal domains in viral class I fusion proteins are conserved targets of broadly neutralizing antibodies (bNAbs), but it has proven difficult to develop vaccines against those targets. The HIV-1 gp41 membrane-proximal external region (MPER) is one such target. Induction of a neutralizing response likely depends on the immunogen having a close-to-native structure. Methods: Native sequence MPER was displayed on genome-reduced bacteria as a coiled-coil homotrimer using a *Haemophilus influenzae* Hia trimeric autotransporter. Vaccine designs incorporated additional features, including trimerization domains to stabilize MPER, tandem MPER repeats to increase antigen valency, and immunomodulatory elements. Antigen exposure was assessed by flow cytometry, antibody responses were evaluated by ELISA, and functional activity was measured using HIV-1 pseudovirus neutralization assays. Results: Trimer stabilization improved MPER exposure, but immunogen visibility alone did not predict neutralization. After three immunizations, neutralizing activity was detected only in the most extensively engineered vaccine, which neutralized tier 2 virus CNE55. After five immunizations, the same vaccine also neutralized the tier 2 virus 25710-2.43. A further design modification that included an extended Hia-derived spacer increased MPER exposure and antibody binding, with neutralization detected against MN.3, X1632_S2_B10, and 25710-2.43 viruses in subsets of animals. Conclusions: As a proof-of-concept, native-sequence MPER can induce detectable, though modest and virus-dependent, HIV-1 neutralizing activity when displayed in a carefully controlled trimeric bacterial surface-display platform. The results show that MPER vaccine performance depends not only on antigen exposure, but also on multimeric organization, immunomodulatory context, and antigen-scaffold geometry. Analogous coiled-coil trimeric bacterial surface display immunogens may inform vaccine development for stem/stalk regions of other Class I fusion protein viruses.

## 1. Introduction

Class I viral fusion proteins from many enveloped viruses, including coronaviruses, influenza viruses, and retroviruses [1], exist as trimers anchored in the viral envelope lipid bilayer. These proteins typically contain membrane-proximal trimeric coiled-coil regions and distal domains involved in receptor binding or membrane fusion. Following activation, they undergo conformational rearrangements that expose hydrophobic fusion peptides, which insert into the host cell membrane and initiate viral envelope–host cell membrane fusion. Antibodies targeting conserved stem/stalk regions can be broadly cross-protective for several viruses [2,3], and anti-stalk antibodies are indicators of protection [4]. However, these regions are often immunosubdominant, and vaccine development targeting these structures has been difficult.

The analogous region of the HIV-1 envelope glycoprotein gp41, the membrane-proximal external region (MPER), is a well-established target of broadly neutralizing antibodies, including 2F5, 4E10, and 10E8 [5,6,7,8]. However, MPER has remained one of the most challenging objectives for HIV vaccine development [9,10,11]. This difficulty arises from intrinsic properties of the region, including shielding by other regions of Env, the requirement that MPER be present in a vaccine immunogen in a close-to-native coiled-coil trimeric structure, partial embedding within the viral membrane, and potential immune tolerance mechanisms [12,13,14]. As a consequence, most MPER-based immunogens—including soluble, multivalent, and trimeric presentation strategies—have elicited weak or non-neutralizing antibody responses [12], emphasizing the need for alternative strategies that present MPER in a carefully controlled, minimally flexible, well-exposed, close-to-native trimeric conformation [15,16]. The induction of significant neutralization in several studies required not only priming with isolated MPER immunogens, but also subsequent challenge infection with live virus [17,18,19,20], highlighting the challenging nature of MPER as a vaccine target [21,22].

Accumulating evidence indicates that epitope exposure and the details of immunogen structure, rather than sequence alone, are critical determinants of MPER immunogenicity [7,23,24,25]. In the native HIV-1 Env protein, MPER is a trimeric coiled-coil structure that is normally obscured by other regions of gp41, but becomes transiently accessible during conformational changes in Env that occur during viral entry [16,26]. An effective MPER immunogen may require re-creating a native MPER structure, including a trimeric coiled-coil structure, ideally adjacent to a lipid bilayer. The vaccine immunogen should be easily accessible to the cells of the immune system, in contrast to the location of MPER in the native Env structure [27,28,29,30], where the rest of Env makes MPER relatively hidden from the immune system. The challenges associated with developing an HIV-1 MPER vaccine are similar to the problems that have troubled the development of vaccines targeting the analogous stem/stalk structures of other class I viral fusion proteins, such as the influenza hemagglutinin and coronavirus spike proteins [4,31,32].

Bacterial surface-display vaccine platforms provide a versatile technology to systematically investigate multiple design strategies. In the Killed Whole-Cell Genome-Reduced Bacteria (KWC/GRB) vaccine platform, antigens are expressed on the surface of GRB by means of a Gram-negative autotransporter. New versions of candidate vaccines can be produced very rapidly and inexpensively, enabling systematic modulation of epitope density, geometry, and accessibility [33,34]. Initial proof-of-concept studies demonstrated that this platform could induce protective immune responses against viral antigens even in the absence of detectable antibody titers, highlighting its capacity to engage multiple immune mechanisms beyond conventional humoral readouts [33].

Subsequent work using the KWC/GRB platform established antigen surface exposure as a critical design parameter for humoral immunity. Using the HIV fusion peptide as a model antigen, Quintero et al. showed that progressive improvements in surface exposure achieved through multimerization, linker optimization, and incorporation of immunomodulatory elements resulted in substantial enhancements in antigen-specific antibody responses [34]. These findings showed that surface accessibility was required to induce a strong humoral immune response following vaccination with KWC/GRB vaccines.

The KWC/GRB approach was extended to MPER using scaffold MPER immunogens expressed using the AIDA-I monomeric autotransporter, leading to the demonstration that a KWC/GRB vaccine could induce the production of MPER-directed neutralizing activity. Carefully engineered production of a scaffolded MPER epitope, notably multimeric immunogens separated by long, rigid alpha helical linkers, along with added immunomodulators, enabled the induction of functional neutralizing antibodies, providing important validation that a KWC/GRB vaccine could induce neutralizing antibodies against a difficult target: MPER [35].

While scaffolded immunogens offer a means to present the immune system with an MPER structure designed to mimic the native structure, an alternative and potentially complementary strategy is to present native sequence MPER in a way that constrains the MPER sequence to assume a close-to-native conformation. Trimeric autotransporters provide an attractive means [36,37,38,39,40,41]. These proteins naturally assemble as homotrimers in the bacterial outer membrane, with a beta barrel consisting of three non-covalently associated elements, and mediate surface display of three passenger domains in an outward-projecting trimeric architecture. The structure of Hia bears a notable resemblance to the stem/stalk/MPER organization of envelope proteins from viruses with class I viral fusion proteins. These structural features make Hia and other members of the trimeric autotransporter subfamily well suited for testing coiled-coil stem/stalk-like immunogen designs [41,42]. The Hia autotransporter provides a stable trimeric display mechanism that can present heterologous antigens in an outward-projecting conformation from the bacterial outer membrane [43,44]. Together, previous findings with the KWC/GRB vaccine platform and the structural features of Hia and other trimeric autotransporters suggest that recombinant KWC/GRB vaccines using trimeric autotransporters may provide a new strategy to induce immune responses against native coiled-coil stem/stalk/MPERs of viruses with class I fusion proteins.

In the present study, we generated a panel of native-sequence MPER vaccines displayed as homotrimers using the Hia trimeric autotransporter. The panel was designed to systematically evaluate how trimer stabilization, antigen valency, tandem-repeat spacing, and immunomodulatory modules influence MPER exposure, antibody binding, antibody induction following vaccination, and resulting neutralizing activity. By integrating structural prediction, monoclonal antibody binding, ELISA, and pseudovirus neutralization assays, this study defines design principles for optimizing native trimeric MPER immunogens within the KWC/GRB platform and may inform vaccine development against other class I viral fusion protein stem/stalk regions.

## 2. Materials and Methods

### 2.1. Vaccine Design

A panel of trimeric MPER vaccine candidates was designed to investigate how antigen architecture, trimeric stabilization, immunomodulatory elements, and antigen valency influence MPER surface exposure, anti-MPER antibody binding, antibody induction, and neutralizing activity. All vaccine designs encoded the native HIV-1 MPER sequence derived from gp41, corresponding to gp41 residues 656–683 (NEQELLELDKWASLWNWFDITNWLWYIK), and were displayed on the bacterial surface using the Hia trimeric autotransporter. This 28-amino-acid sequence is identical to the MPER peptide used as the capture antigen in the ELISA (Section 2.11), ensuring consistency between the displayed immunogen and the antibody binding readout.

To enforce higher-order organization, selected designs incorporated trimerization domains to stabilize MPER as a homotrimer [45,46]. Immunomodulatory elements were included in a subset of designs, including the Pan-DR epitope (PADRE) [34,47,48,49], a non-cognate universal CD4^+^ T-helper epitope, and recombinant Group B streptococcal surface immunogenic protein (rSIP), a TLR2 and TLR4 agonist [34,50,51]. In the vaccine architecture, rSIP was positioned N-terminal to the trimerization and MPER modules, placing it distal to the Hia autotransporter and the bacterial outer membrane relative to the MPER-Hia junction. In addition, duplication of the MPER sequence was introduced in selected candidates to increase MPER antigen valency and potentially enhance avidity for low-affinity B-cell precursors, as previously observed with multimerized non-trimeric immunogens [34,35].

In later designs, additional structural features were introduced to spatially separate duplicated tandem MPER repeats within the trimeric immunogen. Vaccines were generated through six design stages, with new structural elements incorporated at each stage. The designs progressed from a baseline MPER-only candidate to increasingly complex configurations incorporating trimeric stabilization through heterologous trimerization domains, immunomodulatory elements, increased antigen valency through tandem duplication of the MPER sequence, and additional spacing elements to improve MPER accessibility. In design stage 5, an additional Foldon trimerization domain was introduced between the duplicated MPERs to stabilize each MPER unit within the tandem trimeric assembly. In design stage 6, an extended Hia-derived spacer was added to further modulate the distance and accessibility of the MPER tandem array relative to the bacterial outer membrane lipid bilayer.

### 2.2. Three-Dimensional Structure Prediction

Structural models for each vaccine design were generated using AlphaFold2 v2.3.0 in multimer mode to predict homotrimer assemblies [52,53]. For trimeric candidates, three identical polypeptide chains corresponding to the same MPER domain sequence were provided as input to model homotrimer formation. In designs containing duplicated MPER sequences, both MPER copies were encoded within each chain as part of a single continuous polypeptide sequence. Five independent prediction runs were performed per candidate with template search enabled. For each vaccine design, the highest-confidence model, as determined by pLDDT and pTM scores, was selected and visualized using ChimeraX for figure preparation [54,55,56]. Across all designs, per-residue confidence (pLDDT) was highest for the structured scaffold domains, the Hia β-barrel, and the Foldon or Zipper trimerization domains (pLDDT ≈ 70–90) and lower for the flexible functional and accessory elements, including the MPER repeats, the eHIAs spacer, and PADRE (pLDDT ≈ 50–70); rSIP spanned both ranges depending on the subregion. Global and interface confidence metrics were modest overall and decreased progressively with construct complexity, from pTM 0.62 and ipTM 0.65 for the simplest design (MPER alone) to pTM 0.30–0.34 and ipTM 0.31–0.33 for the most elaborate multidomain designs (design stages 5 and 6; Table S1), consistent with the known limitations of AlphaFold-Multimer for large, elongated, multidomain, membrane-associated trimeric assemblies containing intrinsically flexible segments. Notably, the designs with the lowest global confidence were those that elicited detectable neutralization, indicating that model confidence did not predict functional performance. The MPER antigen itself, the functionally critical element, consistently fell in the lower-confidence range across all designs. Structural models were therefore used solely as qualitative, hypothesis-generating design rationale rather than as structural evidence, and functional conclusions were based on the experimental flow cytometry, ELISA, and neutralization data (per-design confidence values are provided in Table S1). Structural predictions were used qualitatively to assess trimeric organization, domain orientation, relative spatial arrangement of MPER copies, and potential steric constraints within the assembly. The Hia barrel-envelope analysis described below was used as an additional operational geometric metric to compare the relative positioning of the Hia-proximal MPER repeat between design stages 5 and 6. No energetic interpretations or experimentally validated structural constraints were inferred from these models.

### 2.3. Hia Barrel-Envelope Structural Analysis

To quantify the relative positioning of the Hia-proximal MPER repeat, a barrel-envelope analysis was performed using the selected AlphaFold2 multimer models for design stages 5 and 6. The proximal MPER repeat corresponded to residues 217–244 in both designs. In design stage 5, Hia was defined as residues 245–308. In design stage 6, eHIAs corresponded to residues 245–252 and Hia was defined as residues 253–316. Chains A, B, and C were analyzed independently. Because the inter-domain arrangement underlying this analysis was predicted with low global and interface confidence (pTM ≈ 0.30–0.34, ipTM ≈ 0.31–0.33 for these designs), barrel-envelope quantities such as residue counts and axial displacements are interpreted as relative, model-based design metrics rather than as accurate structural measurements.

For each predicted trimer, Cα atoms from the Hia region were used to estimate the principal Hia barrel axis by principal component analysis. All non-hydrogen Hia atoms were then projected onto this axis to define the axial span and local radial envelope of the predicted barrel. Each proximal MPER residue was represented by its non-hydrogen atom centroid and classified relative to the Hia envelope. The local radial envelope was estimated using the 95th percentile of Hia radial distances within a ±4.0 Å axial window, with a 1.5 Å radial margin and a 1.0 Å axial margin. Residues within ±0.5 Å of the radial threshold were classified as boundary residues.

This analysis was used as an operational geometric metric to compare local MPER-Hia positioning between predicted models. It was not intended to infer energetic stability, membrane insertion, or experimentally validated residue burial.

### 2.4. Plasmid Construction

**Figure 1:**
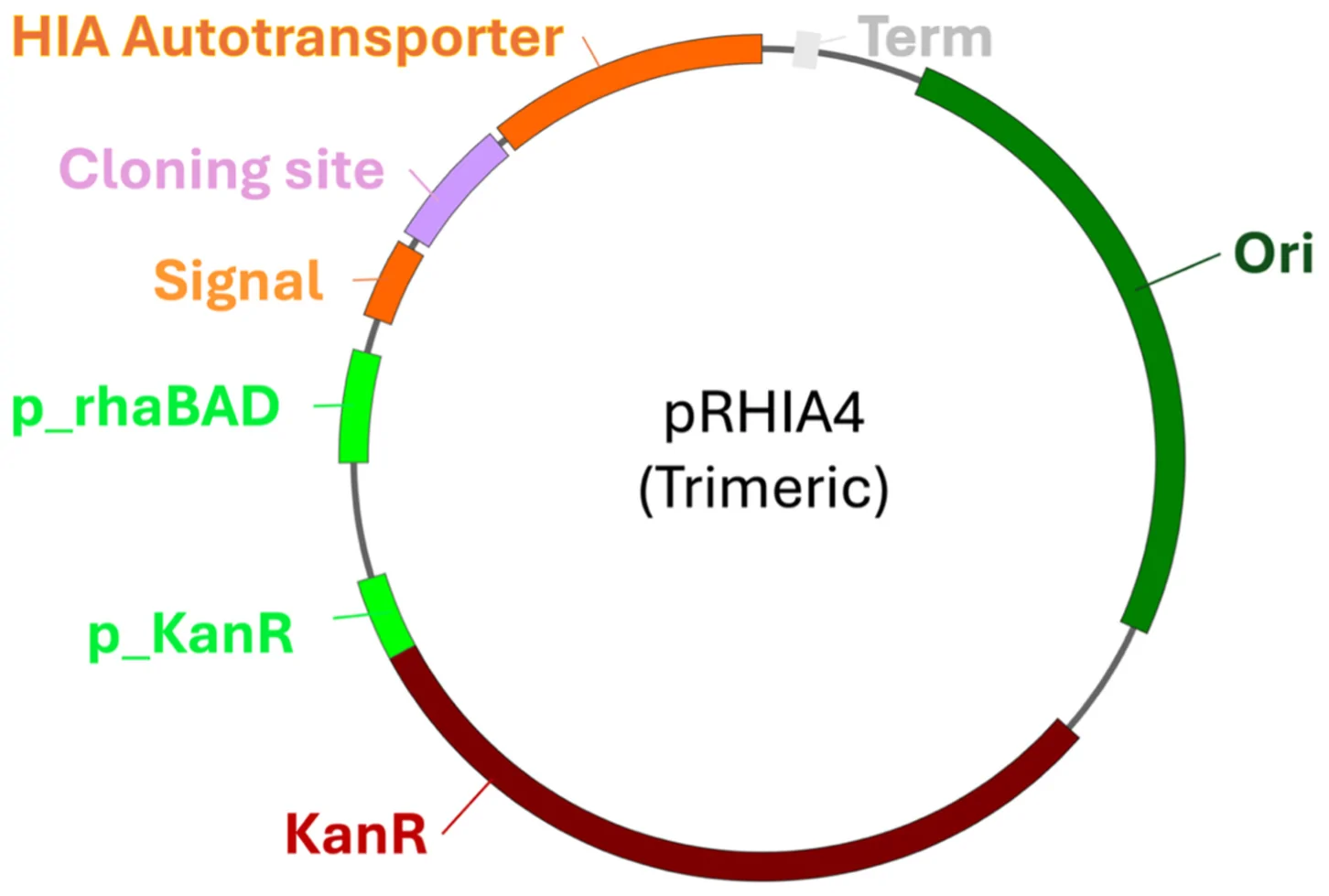
Schematic map of the pRHIA4 trimeric autotransporter expression vector used for construction of MPER vaccine immunogens. The pRHIA4 plasmid contains the rhamnose inducible promoter p_rhaBAD, the kanamycin resistance cassette (KanR), a high copy origin of replication (Ori), a N-terminal signal sequence, a cloning site for insertion of recombinant immunogen sequences, and the *Haemophilus influenzae* adhesin (Hia) trimeric autotransporter cassette for bacterial outer membrane surface display of recombinant proteins. The Hia autotransporter enables presentation of heterologous antigens as trimeric assemblies extending outward from the bacterial outer membrane lipid bilayer. The pRHIA4 sequence has been deposited in GenBank under accession number PZ132917. Abbreviations: Hia, *Haemophilus influenzae* adhesin; Ori, origin of replication; KanR, kanamycin resistance gene; p_rhaBAD, rhamnose inducible promoter.

DNA sequences encoding the trimeric MPER vaccine constructs were commercially synthesized by Twist Bioscience (South San Francisco, CA, USA). Synthesized DNA fragments were cloned into the pRHIA4 expression vector and sequence verified before use. The pRHIA4 plasmid contains a rhamnose-inducible promoter, a high-copy origin of replication, a kanamycin resistance gene, a cloning site for insertion of vaccine immunogen sequences, a N-terminal signal sequence, and the Hia autotransporter cassette, which mediates trimeric surface display of recombinant proteins through insertion into the bacterial outer membrane (Figure 1). The pRHIA4 sequence has been deposited in GenBank under accession number PZ132917.

### 2.5. Bacterial Strain and Transformation

The genome-reduced *Escherichia coli* strain ME5125, with 29.7% of its genome deleted, derived from *E. coli* MG1655, was obtained from Dr. Jun Kato (Tokyo Metropolitan University, Tokyo, Japan) through the National Bioresource Project [57,58]. The strain was routinely cultured in LB broth or on LB agar at 37 °C. Following transformation with pRHIA4-based expression constructs, cultures were maintained in media supplemented with kanamycin (50 µg/mL) for plasmid maintenance. To generate electrocompetent cells, ME5125 cultures were grown overnight at 37 °C, diluted 1:100 into fresh LB medium, and incubated until logarithmic growth was reached, as monitored by OD_600_. Cells were washed sequentially with ice-cold distilled water and ice-cold water containing 10% glycerol, resuspended in water containing 10% glycerol, and electroporated with recombinant pRHIA plasmids using 0.1 cm cuvettes and a Gene Pulser Xcell™ system (Bio-Rad Laboratories, Hercules, CA, USA) at 1.8 kV, 25 µF, and 200 Ω. Following electroporation, cells were recovered for 1 h at 37 °C in SOC medium and plated on LB agar containing kanamycin for colony selection.

### 2.6. Colony PCR Verification

Successful transformation was verified by colony PCR. Three to five kanamycin-resistant colonies from each vaccine design were screened. A sterile pipette tip was used to gently touch each colony, which was then transferred into 5 mL of LB broth containing kanamycin (50 µg/mL) to initiate an overnight starter culture. The same tip was subsequently dipped into a 16 µL PCR reaction prepared using Phusion Flash High-Fidelity PCR Master Mix (Thermo Fisher Scientific, Waltham, MA, USA) and the primer pair pRHIA4 forward (5′-CAGCATATGCACATGGAACA-3′) and pRHIA4 reverse (5′-CGAATAATCACTTTGCCGTTATAGG-3′). PCR cycling conditions were: 95 °C for 2 min, followed by 35 cycles of 95 °C for 45 s, 58 °C for 45 s, and 72 °C for 1 min 20 s, with a final extension at 72 °C for 5 min. Amplicons were resolved on 1.5% agarose/TAE gels and visualized under UV illumination. Colonies yielding amplicons of the expected size were selected for downstream vaccine production. Verified starter cultures were expanded overnight, and glycerol stocks were prepared by mixing cultures 1:1 with sterile 30% glycerol (final concentration 15%) and stored at −80 °C.

### 2.7. Vaccine Production and Chemical Inactivation

Overnight cultures were prepared in LB broth (Thermo Fisher Scientific) supplemented with kanamycin (50 µg/mL) and incubated at 37 °C with shaking at 210 rpm. The following day, cultures were diluted 1:10 into fresh LB medium and grown to an OD_600_ of 0.5–0.6. Protein expression was induced by addition of L-rhamnose (Sigma-Aldrich, St. Louis, MO, USA) to a final concentration of 5 mM and continued for 3 h at 37 °C. Bacterial cells were harvested by centrifugation at 5000× *g* for 30 min at 4 °C and resuspended in Hank’s Balanced Salt Solution (HBSS) (Gibco, Grand Island, NY, USA) containing 0.2% formalin. Chemical inactivation was carried out for 1 h at 37 °C with gentle agitation. Following inactivation, bacterial pellets were washed twice with 1× PBS (Gibco) to remove residual formalin. Final vaccine preparations were resuspended in PBS containing 20% glycerol, normalized to an OD_600_ of 1.0 (corresponding to approximately 8 × 10^8^ bacteria/mL), aliquoted into sterile cryovials, and stored at −80 °C until use.

### 2.8. Post-Inactivation Sterility Testing

Sterility of inactivated vaccine preparations was assessed immediately following the final PBS wash. Triplicate 100 µL aliquots from each preparation were serially diluted (10^0^–10^−4^) and spread onto LB agar plates, which were incubated at 37 °C for 7 days. In parallel, 1 mL of each preparation was inoculated into 10 mL of LB broth and incubated statically at 37 °C for 14 days. Absence of colony formation and turbidity confirmed complete bacterial inactivation.

### 2.9. Flow Cytometry

Formalin-inactivated bacterial suspensions (5 × 10^7^ cells/mL; intact, non-permeabilized whole cells) were blocked in PBS containing 10% fetal bovine serum (FBS; Gibco) for 30 min on ice. Samples were incubated with a serial dilution of the anti-HIV-1 MPER monoclonal antibody 2F5 for 30 min on ice. After washing with PBS supplemented with 2% FBS, cells were stained with Alexa Fluor 488-conjugated goat anti-human IgG (Invitrogen, Thermo Fisher Scientific, Carlsbad, CA, USA) at a dilution of 1:600 for an additional 30 min on ice. Data acquisition was performed using an Attune CytPix Flow Cytometer (Invitrogen), and analyses were conducted with FCS Express 7 software (De Novo Software). Gating was based on forward- and side-scatter parameters. Binding curves were generated from fluorescence values across the 2F5 dilution series and used to estimate EC_50_ values by nonlinear least-squares fitting. EC_50_ estimates were transformed to exposure_score (−log_10_EC_50_). In addition, the integrated flow cytometry signal (AUC(FLOW)) was calculated as the area under the fluorescence–antibody concentration curve. These two metrics capture complementary aspects of surface 2F5 reactivity: the exposure_score reflects the sensitivity (potency) of binding—the antibody concentration required for half-maximal binding, such that higher values indicate a detectable epitope at lower antibody concentrations—whereas AUC(FLOW) reflects the integrated magnitude of binding across the full titration. Both are relative, composite proxies for the amount of surface-available MPER, reflecting surface expression, display efficiency, and epitope accessibility, and neither provides an absolute measure of antigen surface density. Experimental controls included untransformed ME5125 bacteria, isotype controls, secondary-only controls, and PBS blanks. All flow cytometry experiments were performed at the University of Virginia Flow Cytometry Core Facility.

### 2.10. Mouse Immunization

Quasi-outbred HET3 mice (F1[CByB6F1 × B6C3F1]; Jackson Laboratory, stock #036603) were obtained at six weeks of age and acclimated for at least 7 days under specific-pathogen-free conditions with a 12 h light/dark cycle and ad libitum access to food and water.

Animals were block-allocated into experimental groups corresponding to each vaccine candidate, along with control groups receiving either PBS or formalin-inactivated untransformed ME5125 bacteria. Each group consisted of five mice (three females and two males). Immunizations were administered intramuscularly in the quadriceps using 5 × 10^9^ inactivated bacteria in 50 µL PBS per dose.

All vaccine candidates were initially evaluated using a three-dose immunization regimen at weeks 0, 3, and 6. Following detection of neutralizing activity for design stage 5, this vaccine and the subsequently generated design stage 6/eHIAs derivative were evaluated using an extended five-dose regimen at weeks 0, 3, 6, 9, and 12. Blood samples were collected prior to immunization and at subsequent time points. At week 15, terminal cardiac exsanguination was performed under deep surgical anesthesia. Plasma was isolated by centrifugation and stored at −80 °C until analysis. Animals were monitored throughout the study for signs of local reactogenicity or systemic adverse effects.

### 2.11. Enzyme-Linked Immunosorbent Assay (ELISA)

High-binding 96-well plates (Thermo Fisher Scientific, Waltham, MA, USA) were coated overnight at 4 °C with recombinant HIV-1 MPER peptide corresponding to gp41 residues 656–683 (sequence: NEQELLELDKWASLWNWFDITNWLWYIK; Biomatik, Wilmington, DE, USA) at a final concentration of 1 µg/mL in phosphate-buffered saline (PBS; pH 7.4). Plates were washed three times with TBST (Tris-buffered saline containing 0.05% Tween-20; Thermo Fisher Scientific) and blocked for 1 h at room temperature with 3% bovine serum albumin (BSA; Sigma-Aldrich) prepared in TBST. Mouse plasma samples were serially diluted from 1:50 to 1:102,400 using a four-fold dilution series in 1% BSA/TBST, and each dilution was added to the plates in duplicate. After incubation for 1 h at room temperature, plates were washed and bound IgG was detected using HRP-conjugated goat anti-mouse IgG (Thermo Fisher Scientific) diluted 1:5000 in 1% BSA/TBST and incubated for 1 h at room temperature. Following additional wash steps, plates were developed using TMB substrate (Thermo Fisher Scientific) and reactions were stopped with 1 N sulfuric acid. Absorbance was measured at 450 nm using an accuSkan FC microplate reader (Fisher Scientific, Hampton, NH, USA). Each assay included an eight-point standard curve generated with the 2F5 monoclonal antibody (starting concentration 1 µg/mL with three-fold serial dilutions) to verify assay performance and enable calculation of relative antibody units. ELISA titration curves were fitted using a four-parameter logistic model, and antibody binding responses were quantified as the area under the curve (AUC). Negative controls included wells without antigen, secondary-only controls, pre-immune plasma pools, and plasma from mice immunized with formalin-inactivated untransformed *E. coli* prepared in parallel with vaccine strains.

### 2.12. Neutralization Assays

Neutralization assays were performed at Duke University using HIV-1 Env pseudoviruses (25710-2.43, CNE55, MN.3, and X1632_S2_B10) and TZM-bl cells [59,60], following standard operating procedures routinely used for evaluation of vaccine-elicited neutralizing antibody responses. Mouse plasma samples were heat-inactivated at 56 °C for 15 min and serially diluted in cell culture medium. Diluted plasma samples were incubated with pseudovirus for 1 h at 37 °C and subsequently added to TZM-bl cells. After approximately 48 h, luciferase activity was measured. Neutralization titers were expressed as ID_50_ and ID_80_ values, defined as the reciprocal plasma dilutions at which relative luminescence units (RLUs) were reduced by 50% or 80%, respectively, compared with virus-only control wells after subtraction of background RLUs from cell-only controls. Samples that did not achieve 50% inhibition within the tested dilution range were reported as ID_50_ below the lowest dilution tested. Virus-specific assay positivity ID_50_ cutoffs were defined as the 90th percentile of ID_50_ values obtained from all baselines (pre-immune) mouse plasma samples and were applied as follows: 84.2 for CNE55, 121 for X1632_S2_B10, 63 for 25710-2.43, and 45 for MN.3. A post-immunization sample was classified as neutralization-positive only when the post-immunization ID50 was greater than the virus-specific cutoff and the corresponding baseline (pre-immune) ID50 did not exceed that cutoff. For the MN.3 pseudovirus, whose 90th-percentile cutoff (45) coincided with the assay starting dilution, this criterion identifies samples whose post-immunization ID50 rose above the assay floor relative to a baseline at the floor. The 90th-percentile baseline threshold was selected as a conservative, pre-specified criterion anchored to the empirical distribution of pre-immune (baseline) ID_50_ values: defining positivity relative to the top decile of background reactivity for each pseudovirus limits the baseline false-positive rate to approximately 10% per virus, and the additional requirement that the matched pre-immune sample from the same animal fall below the cutoff further reduces spurious calls. Testing each post-immunization sample against its matched pre-immune control accounts for low-level, sample-specific non-specific activity, which is particularly relevant for plasma. Assays were performed under the standardized operating procedures of the Duke laboratory, routinely used for evaluation of vaccine-elicited neutralizing antibody responses, with virus-only and cell-only control wells included in every assay. Each plasma sample was analyzed individually rather than pooled, with biological replicates corresponding to individual animals (*n* = 5 per group), and each dilution series was tested in duplicate wells per the standardized protocol.

The HIV-1 pseudovirus panel included MN.3, a tier 1A virus, and the tier 2 viruses 25710-2.43, CNE55, and X1632_S2_B10. Thus, three of the four strains correspond to more difficult-to-neutralize tier 2 viruses, providing a stringent panel for evaluating virus-dependent neutralization activity across pseudoviruses of differing sensitivity.

### 2.13. Statistical Analysis

All statistical analyses were performed in R v4.4.1 using the packages tidyverse, ggplot2, psych, corrplot, AER, lmtest, car, broom, and pROC. The complete analysis workflow, including data preprocessing, statistical testing, and figure generation, was implemented using custom R scripts developed by the authors with assistance from ChatGPT (OpenAI, GPT-5 model). Fully annotated scripts and processed datasets are provided in the Supplementary Materials (File S1, R script; File S2, processed datasets) to ensure reproducibility.

Antibody binding responses were quantified using AUC (ELISA) values calculated from serial dilution curves fitted with a four-parameter logistic regression model. AUC values were computed at the individual animal-level and used for all downstream statistical comparisons. Flow-cytometry titration curves obtained with the monoclonal antibody 2F5 were fitted using nonlinear least-squares regression to estimate EC_50_ values, which were transformed into an exposure_score (−log_10_EC_50_) used as a metric of MPER surface detectability by 2F5. Because this signal reflects the combined contributions of surface expression level, display efficiency, and epitope accessibility, we interpret it as a composite measure of surface-available MPER rather than of epitope accessibility alone. In parallel, the integrated flow-cytometry signal (AUC (FLOW)) was calculated as the area under the fluorescence–antibody concentration curve; like the exposure_score, AUC (FLOW) is interpreted as a relative, composite proxy for surface-available MPER rather than an absolute measure of antigen density. Comparisons of antibody binding responses between vaccine groups and between successive design stages were conducted using the Wilcoxon rank-sum test, given the small group sizes and the absence of assumptions of normality. Effect sizes were quantified using Cliff’s delta, and 95% confidence intervals were estimated by bootstrap resampling. Stepwise comparisons between consecutive design stages were performed using animal-level AUC (ELISA) values to isolate the effect of individual architectural modifications. To assess the impact of trimerization strategy, Foldon-based and Zipper-based candidates were compared within design stages where both architectures were present, again using Wilcoxon rank-sum tests and Cliff’s delta to quantify effect magnitude. Neutralization data were analyzed per pseudovirus. For descriptive plotting and log10 transformation, ID50 values below the assay detection limit were assigned the corresponding virus-specific cutoff value. Categorical neutralization positivity was determined independently using the virus-specific cutoff and baseline rule described above. Neutralization outcomes were treated as categorical at the vaccine-level for interpretation of functional activity, given that detectable neutralization was restricted to a single vaccine design under the three-dose regimen. Associations between antigen exposure metrics (exposure_score and AUC (FLOW)) and antibody binding magnitude (mean AUC (ELISA) per vaccine) were evaluated using both Pearson correlation coefficients to assess linear relationships and Spearman rank correlations to capture monotonic trends. These analyses were performed at the vaccine level, using mean ELISA AUC values to avoid pseudo-replication. Unless otherwise stated, statistical comparisons of AUC (ELISA) across design stages were performed using plasma collected after the third immunization in the three-dose regimen. Unless otherwise stated, data are presented as mean ± SEM, and statistical significance was defined as *p* < 0.05. Given the exploratory nature of this study and the limited sample sizes inherent to animal vaccination experiments, effect sizes and confidence intervals were emphasized alongside *p*-values to support quantitative interpretation. Because analyses were exploratory and hypothesis-generating within a sequential design-build-test-learn framework, and because group sizes were small, we emphasized effect sizes (Cliff’s delta) and 95% confidence intervals rather than significance thresholds, and we did not apply corrections for multiple comparisons; reported *p*-values are therefore descriptive rather than confirmatory. We deliberately avoided multiplicity adjustment in this setting because, with small samples and an exploratory objective, such corrections would substantially inflate the type II error rate and risk discarding genuine signals that merit follow-up. For each stage in the DBTL process, our pre-established hypothesis was that, for each DBTL cycle, the subsequent design would be better than the prior one, a study design motivating the pairwise statistical comparison. Correlation analyses across vaccine designs (Section 3.11) were similarly exploratory and are limited by the small number of designs available.

### 2.14. Ethics, Animal Welfare, and Biosafety

All animal procedures were conducted in accordance with the Guide for the Care and Use of Laboratory Animals and were approved by the University of Virginia Animal Care and Use Committee (protocol 41101224; approval date 5 November 2024; Unique ID 7018391). Recombinant DNA work and handling of formalin-inactivated bacterial vaccine preparations were performed under BSL-2 conditions approved by the University of Virginia Institutional Biosafety Committee (IBC protocol 4276-15).

## 3. Results

### 3.1. Vaccine Design, Composition, and Dataset Used for Analysis

We pursued a stepwise vaccine design strategy to evaluate how successive modifications of trimeric native coiled-coil MPER vaccine architecture affected antigen visibility, antibody binding, and neutralizing activity. Vaccines were grouped into design stages, where each stage represents a defined structural configuration evaluated experimentally.

Design features incorporated at successive stages were selected based on findings from prior KWC/GRB studies demonstrating that antigen surface exposure, multimeric organization, and immunomodulatory elements influence antibody responses and functional immunogenicity. Earlier studies using HIV fusion peptide and scaffolded MPER immunogens established antigen accessibility as an important design parameter and demonstrated that structural stabilization of MPER could enable induction of neutralizing activity [34,35].

The present study extended these observations by progressively modifying native-sequence MPER immunogens through incorporation of heterologous trimerization domains, immunomodulatory elements, tandem MPER duplication, and additional spacing elements intended to alter MPER presentation within the trimeric assembly.

Table 1 summarizes the vaccine components included in this study, detailing the presence or absence of trimerization domains, immunomodulatory elements, MPER valency modifications, and spacing elements. All vaccines evaluated across the study are listed in Table 2 and are referenced below.

All experimental readouts presented below were analyzed using the complete dataset summarized in Table 3, which includes flow cytometry measurements and neutralization results for all vaccines.

### 3.2. Design Stage 0: MPER-Only Baseline

**Figure 2:**
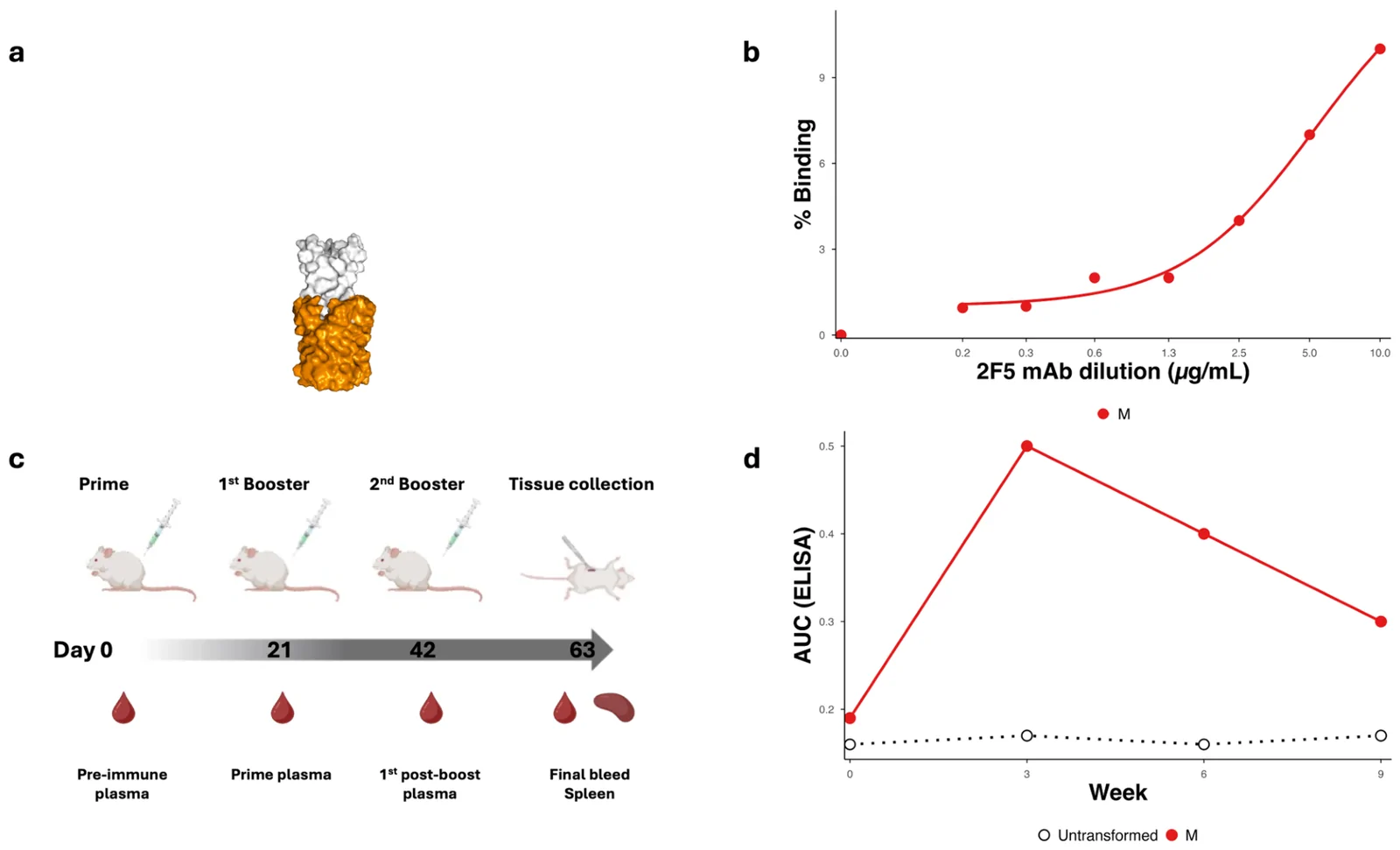
Baseline characterization of the MPER-only vaccine (design stage 0). (**a**) Predicted three-dimensional structure of the MPER-only candidate displayed via the trimeric Hia autotransporter. The MPER is shown in white, and the Hia β-barrel/translocator domain is shown in orange. (**b**) Flow cytometry analysis of surface-displayed MPER using the broadly neutralizing monoclonal antibody 2F5. Formalin-inactivated bacteria expressing MPER alone were incubated with increasing concentrations of 2F5, and binding is shown as percent of positive cells relative to background. Data illustrate weak but detectable MPER accessibility across the antibody dilution range. (**c**) Immunization and sampling schedule for the three-dose regimen. Mice were immunized intramuscularly at day 0 (prime), day 21 (first booster), and day 42 (second booster). Plasma samples were collected prior to immunization and after each dose, and terminal tissue collection was performed at day 63. (**d**) Longitudinal MPER-specific antibody responses measured by ELISA and expressed as area under the curve (AUC). Red symbols correspond to mice immunized with the MPER-only vaccine, and black symbols correspond to mice immunized with formalin-inactivated untransformed bacteria. Antibody responses were low and transient, with no sustained increase following boosting. Abbreviations: MPER, membrane-proximal external region; AUC, area under the curve; ELISA, enzyme-linked immunosorbent assay; mAb, monoclonal antibody.

We first evaluated a baseline vaccine expressing trimeric MPER alone using the Hia trimeric autotransporter. Structural prediction suggested that, in the absence of dedicated trimerization domains, MPER is presented without additional structural stabilization beyond the Hia scaffold itself (Figure 2a). In addition, expression of a single MPER alone may provide limited control over the lateral presentation of the MPER shaft, where several broadly neutralizing antibodies bind.

Flow cytometry analysis revealed low MPER surface exposure, with weak binding signals across dilution ranges (Figure 2b). Consistent with these findings, mice immunized with the MPER-only vaccine using the three-dose regimen exhibited detectable anti-MPER antibody responses by ELISA, but these responses showed only minimal increases after repeated immunization (Figure 2c,d). Neutralization assays performed using sera from these animals showed no detectable activity against any of the tested HIV-1 pseudoviruses.

### 3.3. Design Stage 1: Stabilization of MPER Coiled-Coil Homotrimers Using Trimerization Domains

**Figure 3:**
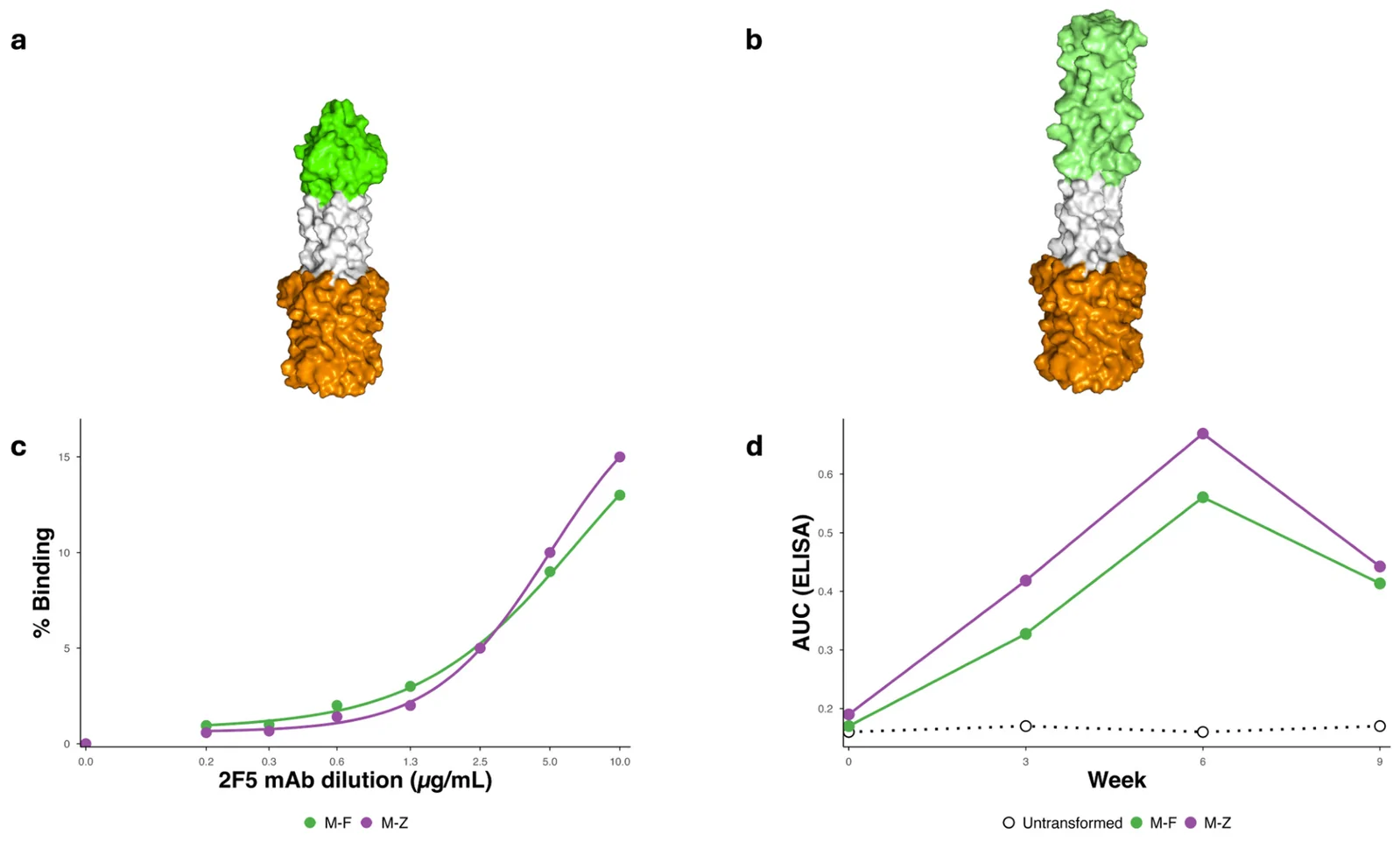
Stabilization of MPER homotrimers using trimerization domains (design stage 1). (**a**) Predicted three-dimensional structure of the MPER candidate stabilized with a Foldon trimerization domain. The MPER is shown in white, the Foldon trimerization domain is shown in dark green, and the Hia β-barrel/translocator domain is shown in orange. Structural prediction illustrates enforced trimeric organization of MPER compared with the MPER-only candidate. (**b**) Predicted three-dimensional structure of an alternative trimerization strategy. As in panel (**a**), MPER is shown in white, the Zipper trimerization domain in light green, and the Hia beta barrel region is shown in orange, highlighting differences in domain length and spatial arrangement between trimerization designs. (**c**) Flow cytometry analysis of surface-displayed MPER stabilized with trimerization domains using the broadly neutralizing monoclonal antibody 2F5. Formalin-inactivated bacteria expressing MPER with Foldon-based (M-F) or Zipper-based (M-Z) trimerization domains were incubated with increasing concentrations of 2F5, and binding is shown as percent positive cells. Trimerization strategies increased 2F5 surface binding (MPER surface detectability) relative to the MPER-only candidate. (**d**) Longitudinal MPER-specific antibody responses measured by ELISA and expressed as area under the curve (AUC) following immunization with trimerized MPER vaccines. Green symbols correspond to Foldon-based candidates (M-F), purple symbols to Zipper-based candidates (M-Z), and black symbols to formalin-inactivated untransformed bacteria. Trimeric stabilization enhanced antibody binding responses relative to controls, with comparable kinetics between trimerization strategies. Abbreviations: MPER, membrane-proximal external region; AUC, area under the curve; ELISA, enzyme-linked immunosorbent assay; mAb, monoclonal antibody.

To assess whether additional trimer stabilization could improve the performance of Hia-displayed MPER immunogens, we evaluated vaccines incorporating either Foldon or Zipper trimerization domains [45,46]. Structural modeling indicated that adding either of the trimerization domains should help stabilize MPER designs compared with baseline MPER-only design (Figure 3a,b).

Flow cytometry binding curves demonstrated increased antigen exposure compared with design stage 0. Distinct binding profiles were observed between vaccines containing different trimerization domains (Figure 3c). ELISA AUC values were higher than those observed for the MPER design lacking an additional trimerization domain (Figure 3d). Neutralization assays performed after three immunizations did not detect neutralizing activity for any stage 1 design.

### 3.4. Design Stage 2: Addition of rSIP to Stabilized MPER Candidates

**Figure 4:**
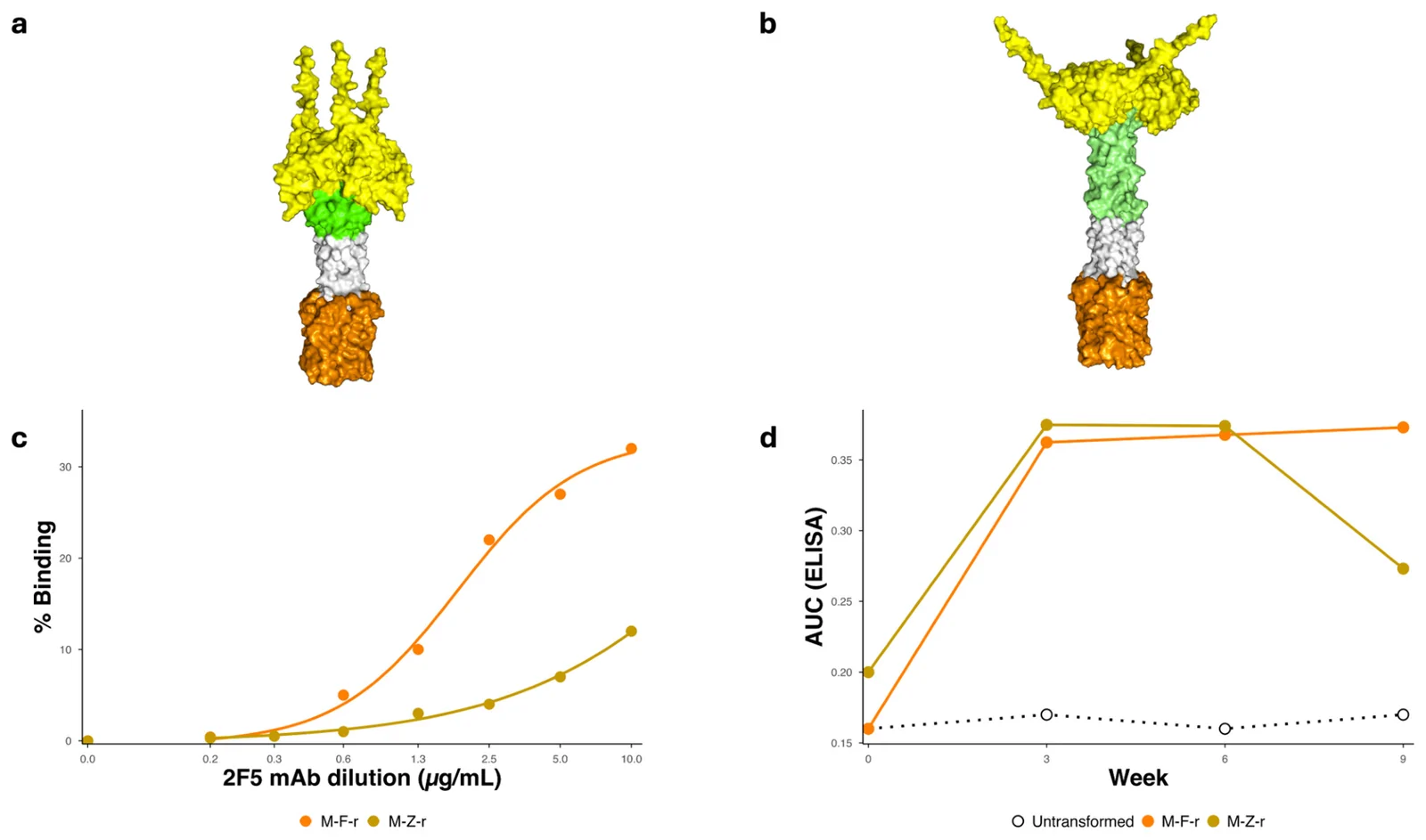
Effect of rSIP incorporation on stabilized MPER homotrimer vaccines (design stage 2). (**a**) Predicted three-dimensional structure of M-F-r vaccine incorporating a trimerization domain together with the recombinant Group B streptococcal surface immunogenic protein (rSIP). MPER is shown in white, the trimerization domain Foldon in dark green, rSIP in yellow, and the Hia β-barrel/translocator domain in orange. The modeling suggested that the rSIP immunomodulators would be present N-terminal to the trimerization and MPERs and that MPER would assume a coiled-coil trimeric organization. (**b**) Predicted three-dimensional structure of M-Z-r vaccine containing rSIP, highlighting differences in rSIP orientation and overall spatial arrangement relative to the MPER homotrimer. Color coding is as in panel (**a**) except for the trimeric domain Zipper in light green. (**c**) Flow cytometry analysis of surface-displayed MPER incorporating rSIP, measured using the broadly neutralizing monoclonal antibody 2F5. Formalin-inactivated bacteria expressing Foldon-based (M-F-r, orange) or Zipper-based (M-Z-r, yellow) trimeric MPER vaccines were incubated with increasing concentrations of 2F5, and binding is reported as percent positive cells. Incorporation of rSIP altered the magnitude and shape of the 2F5 binding curves relative to trimeric candidates lacking rSIP. (**d**) Longitudinal MPER-specific antibody responses measured by ELISA and expressed as area under the curve (AUC) following immunization with rSIP-containing trimeric MPER vaccines. Orange symbols correspond to Foldon-based candidates with rSIP (M-F-r), yellow symbols to Zipper-based candidates with rSIP (M-Z-r), and black symbols to formalin-inactivated untransformed bacteria. Antibody binding responses increased after immunization but showed differences in magnitude and durability between vaccines. Abbreviations: MPER, membrane-proximal external region; rSIP, recombinant Group B streptococcal surface immunogenic protein; AUC, area under the curve; ELISA, enzyme-linked immunosorbent assay; mAb, monoclonal antibody.

Design stage 2 examined MPER candidates containing an additional trimerization domain together with the incorporation of the rSIP immunomodulator (TLR-2 and -4 agonist). We hypothesized that adding rSIP to the MPER immunogen would enhance immune responses, as we had previously observed with a monomeric immunogen [34,35]. Structural modeling of these candidates revealed that the overall homotrimer organization of MPER was maintained, while rSIP was predicted to be positioned outside the MPER, with the MPER predicted to be accessible (Figure 4a,b).

Binding profiles obtained by flow cytometry showed altered antigen exposure relative to design stage 1 (Figure 4c). At the vaccine-level, both exposure_score and AUC (FLOW) indicated that several candidates in this design stage maintained or increased MPER surface detectability compared with earlier stages. Antibody binding measured by ELISA yielded AUC values comparable to those observed for design stages 0 and 1 vaccines (Figure 4d). Neutralization assays performed after three immunizations did not detect neutralizing activity for any design stage 2 vaccine.

### 3.5. Design Stage 3: Incorporation of PADRE into rSIP-Containing MPER Vaccines

**Figure 5:**
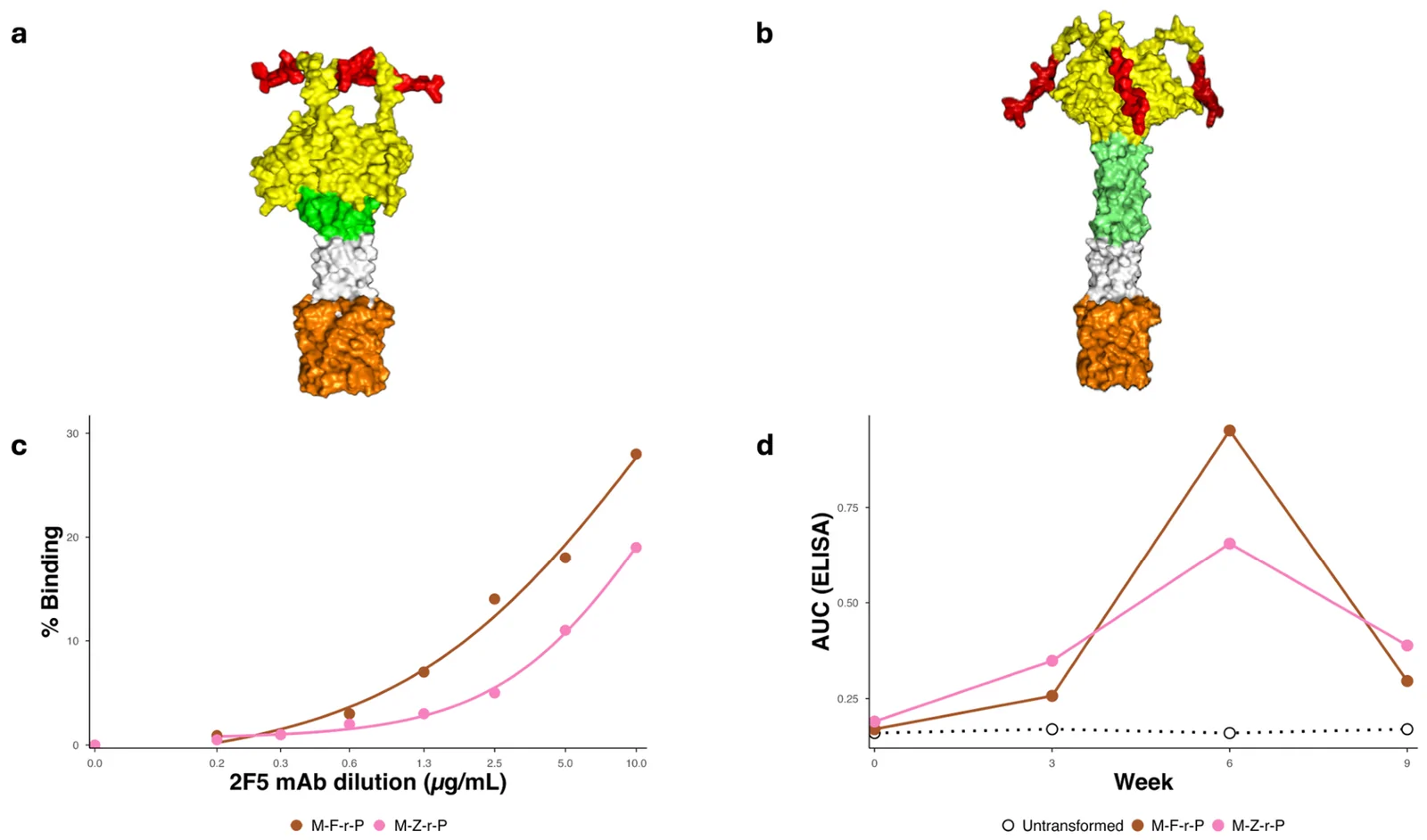
Effect of PADRE incorporation into rSIP-containing trimeric MPER vaccines (design stage 3). (**a**) Predicted three-dimensional structure of M-F-r-P vaccine incorporating a trimeric domain Foldon, rSIP, and the Pan-DR epitope (PADRE). MPER is shown in white, the trimerization domain Foldon in dark green, rSIP in yellow, PADRE in red, and the Hia β-barrel/translocator domain in orange. The model shows preservation of the MPER coiled-coil homotrimer with PADRE positioned distal to the MPER. (**b**) Predicted three-dimensional structure of M-Z-r-P vaccine, highlighting differences in PADRE orientation and overall spatial organization relative to MPER. Color coding is as in panel (**a**) except for the trimerization domain Zipper in light green. (**c**) Surface display of MPER by PADRE-containing vaccines was assessed by flow cytometry using the broadly neutralizing monoclonal antibody 2F5. Formalin-inactivated bacteria expressing Foldon-based (M-F-r-P, brown) or Zipper-based (M-Z-r-P, pink) trimeric MPER candidates were incubated with increasing concentrations of 2F5. Binding is reported as percent positive cells. Incorporation of PADRE increased the magnitude of 2F5 binding compared with corresponding rSIP-containing candidates lacking PADRE. (**d**) Longitudinal MPER-specific antibody responses measured by ELISA and expressed as area under the curve (AUC) following immunization with PADRE-containing trimeric MPER vaccines. Brown symbols correspond to Foldon-based candidates with rSIP and PADRE (M-F-r-P), pink symbols to Zipper-based candidates with rSIP and PADRE (M-Z-r-P), and black symbols to formalin-inactivated untransformed bacteria. Antibody binding responses peaked after the second booster and declined modestly by the final time point. Abbreviations: MPER, membrane-proximal external region; PADRE, Pan-DR epitope; rSIP, recombinant Group B streptococcal surface immunogenic protein; AUC, area under the curve; ELISA, enzyme-linked immunosorbent assay; mAb, monoclonal antibody.

While the vaccines of design stage 1 induced the production of anti-MPER antibodies, the amount of antibody induced was relatively low. We therefore added immunomodulatory sequences that aimed to enhance the immune responses against the MPER. In design stage 3, PADRE was incorporated into MPER candidates that already included rSIP and an additional trimerization domain. Computational models indicated that MPER chains remained organized in a stabilized homotrimer coiled-coil configuration, with PADRE positioned away from the MPERs and the MPER remaining accessible (Figure 5a,b).

Flow cytometry analyses showed antigen exposure equal to or greater than that of earlier design stages (Figure 5c), with exposure_score and AUC (FLOW) values comparable to, or higher than, those measured for related candidates in design stage 2, suggesting that addition of PADRE did not adversely affect MPER accessibility. ELISA AUC measurements were similar to those obtained for previous design stages (Figure 5d). Neutralization assays conducted after three immunizations did not reveal detectable neutralizing activity for design stage 3 candidates.

### 3.6. Design Stage 4: Increased MPER Valency with Stabilized Homotrimer Immunogens

Design stage 4 evaluated vaccines containing tandem duplicated MPER sequences within the established coiled-coil homotrimer framework. We hypothesized that increasing MPER valency could enhance immunogenicity through several complementary mechanisms. First, tandem MPER repeats would increase the antigen dose presented on the bacterial surface. Second, repeated MPER units, if appropriately separated and stabilized, could increase avidity for low-affinity B-cell precursors [62,63]. These features, together with the inherent danger signal provided by a particulate immunogen [64], PADRE-mediated T-cell help, and high local peptide density [65], could support stronger B-cell activation, germinal center entry [66], affinity maturation [67], with development of the desired antibody response.

**Figure 6:**
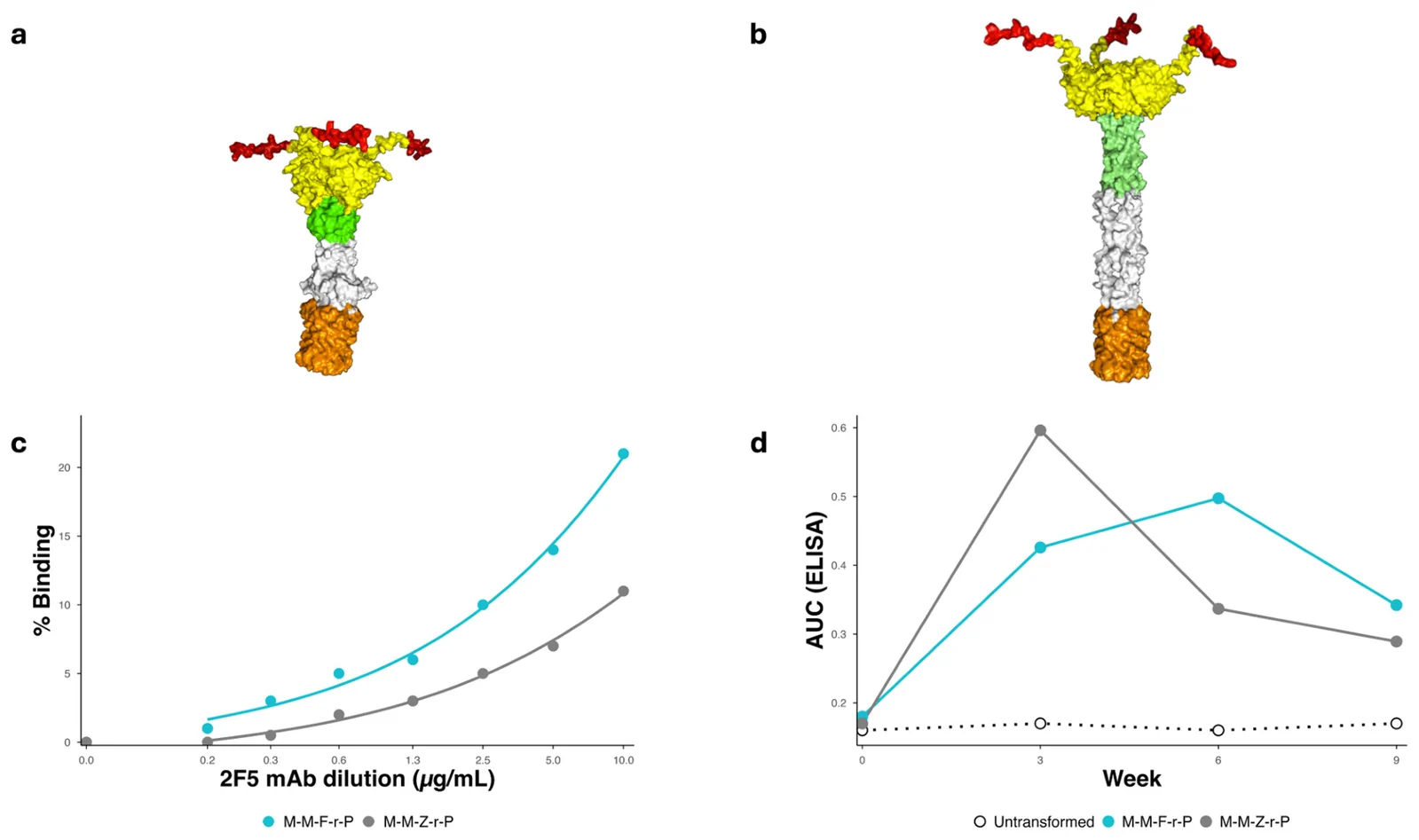
Increased MPER valency within stabilized homotrimer vaccines (design stage 4). (**a**) Predicted structure of the M-M-F-r-P vaccine design containing tandem duplicated MPERs without additional spacing elements between MPER repeats. Structural prediction suggested close spatial proximity between adjacent MPERs within the trimeric assembly and imperfect maintenance of the MPER native coiled-coil structure. MPER is shown in white, the Foldon trimerization domain in dark green, rSIP in yellow, PADRE in red, and the Hia translocator domain in orange. (**b**) Predicted structure of the M-M-Z-r-P vaccine design containing tandem duplicated MPERs stabilized by a leucine zipper trimerization domain. Structural prediction suggested partial preservation of tandem MPER organization, although adjacent MPER repeats remained in close spatial proximity. Color coding is as in panel (**a**), except that the Zipper trimerization domain is shown in light green. (**c**) Flow cytometry analysis of MPER surface exposure measured using the broadly neutralizing monoclonal antibody 2F5. Formalin-inactivated bacteria expressing tandem duplicated MPERs stabilized with either a Foldon trimerization domain (M-M-F-r-P, cyan) or a leucine zipper trimerization domain (M-M-Z-r-P, gray) were incubated with increasing concentrations of 2F5, and binding is reported as percent positive cells. (**d**) Longitudinal MPER-specific antibody binding measured by ELISA and expressed as area under the curve (AUC) following immunization with duplicated MPER vaccines. Cyan symbols correspond to M-M-F-r-P, gray symbols correspond to M-M-Z-r-P, and black symbols correspond to formalin-inactivated untransformed bacteria. Plasma was collected at baseline and at weeks 3, 6, and 9 after immunization, and ELISA AUC values are plotted over time. Abbreviations: MPER, membrane proximal external region; rSIP, recombinant Group B streptococcal surface immunogenic protein; PADRE, Pan-DR epitope; AUC, area under the curve; ELISA, enzyme-linked immunosorbent assay; mAb, monoclonal antibody.

Structural prediction suggested that tandem duplication of MPER could be accommodated within the trimeric assembly and that the stacked tandem MPERs were likely to be surface-exposed. However, the predicted structures also indicated that close spatial proximity between adjacent MPER repeats could influence the organization of the tandem MPER coiled-coil structure, particularly in the M-M-F-r-P design (Figure 6a,b). These observations suggested that simple duplication of the MPER alone may not be sufficient to promote optimal presentation of tandem MPER repeats within the trimeric assembly.

Based on these structural predictions, we hypothesized that additional spacing or stabilization elements might be required to better separate adjacent MPER repeats and preserve a more favorable trimeric presentation. This rationale motivated the subsequent design stages incorporating an additional Foldon trimerization domain between tandem MPERs.

Surface expression and accessibility of MPER were confirmed by flow cytometry binding curves for all vaccines evaluated at this stage (Figure 6c). AUC (FLOW) values were maintained or showed modest increases relative to design stage 3 with the presence of tandem duplicated MPERs. However, the increase in surface exposure was less than might be expected from duplication of the MPER sequence alone, suggesting that tandem MPER duplication did not necessarily translate into proportionally improved epitope accessibility. These observations were consistent with the structural predictions indicating close spatial proximity between adjacent MPERs within the trimeric assembly and imperfect maintenance of the native trimeric MPER coiled-coil structure over the length of the expressed protein.

ELISA AUC values remained within the range observed for earlier design stages (Figure 6d), indicating that tandem MPER duplication alone did not substantially improve antibody binding responses relative to previous vaccine configurations. Neutralization assays performed after three immunizations did not detect neutralizing activity for any design stage 4 vaccine. Together, these findings suggested that increased MPER valency alone was insufficient to improve functional immunogenicity and supported the need for additional structural elements to better spatially organize tandem MPER repeats and promote the maintenance of the desired native coiled-coil structure of the MPERs within the trimeric assembly.

### 3.7. Design Stage 5: Tandem MPER Repeats Stabilized by an Intervening Foldon Trimerization Domain

**Figure 7:**
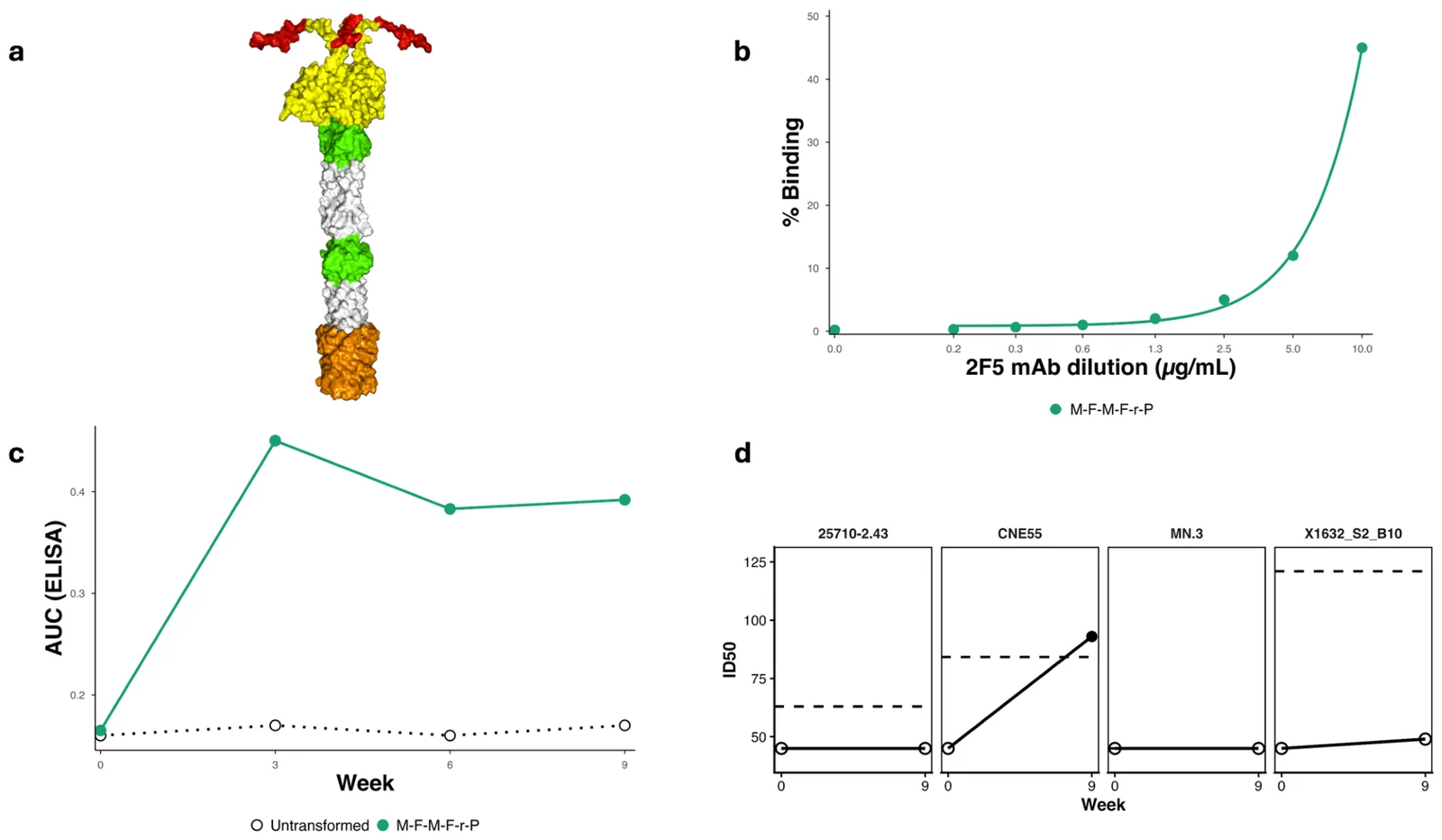
Stabilization of tandem MPER repeats with an intervening Foldon trimerization domain further enhances anti-MPER immune responses to yield vaccines that induce neutralization (design stage 5). (**a**) Predicted three-dimensional structure of the most extensively engineered MPER vaccine design incorporating duplicated MPER sequences separated by trimeric domain Foldon within a stabilized homotrimer assembly. MPER is shown in white, trimeric domains Foldon in dark green, rSIP in yellow, PADRE in red, and the Hia β-barrel/translocator domain in orange. The predicted structure demonstrates increased spatial separation between duplicated MPER repeats relative to design stage 4, while preserving overall trimeric organization. (**b**) Flow cytometry analysis of MPER surface exposure measured using the broadly neutralizing monoclonal antibody 2F5. Formalin-inactivated bacteria expressing the design stage 5 vaccine were incubated with increasing concentrations of 2F5, and binding is reported as percent positive cells, demonstrating enhanced MPER surface detectability relative to earlier design stages. (**c**) Longitudinal MPER-specific antibody binding measured by ELISA and expressed as area under the curve following immunization with the design stage 5 vaccine. Turquoise symbols represent M-F-M-F-r-P and open black symbols represent formalin-inactivated untransformed bacteria. Plasma was collected at baseline and at weeks 3, 6, and 9, corresponding to the three-dose immunization regimen. (**d**) Neutralization activity induced by the design stage 5 vaccine measured against a panel of HIV-1 Env pseudoviruses. Neutralization was assessed using pooled plasma from immunized animals, and titers are shown as ID50 values for 25710-2.43, CNE55, MN.3, and X1632_S2_B10 at baseline and after three immunizations. Each line represents the pooled plasma for a given virus, and dashed horizontal lines indicate virus-specific assay positivity cutoffs. Filled black circles connected by black lines denote a neutralization-positive result, in which the pooled plasma ID50 exceeded the virus-specific cutoff, whereas open circles connected by gray lines denote pools that remained below the cutoff. Neutralizing activity was detected exclusively against the tier 2 virus CNE55 following three immunizations, whereas no neutralization was observed for the remaining viruses. Abbreviations: MPER, membrane proximal external region; rSIP, recombinant Group B streptococcal surface immunogenic protein; PADRE, Pan-DR epitope; AUC, area under the curve; ELISA, enzyme-linked immunosorbent assay; mAb, monoclonal antibody; ID50, reciprocal plasma dilution resulting in 50 percent neutralization.

Because design stage 4 vaccines containing tandem MPER repeats did not induce neutralizing activity and structural predictions suggested close spatial proximity between adjacent MPER modules with imperfect maintenance of the desired native coiled-coil MPER, we hypothesized that each MPER repeat required additional stabilization and separation within the trimeric assembly. Design stage 5 therefore introduced an additional Foldon domain between the duplicated MPER sequences to separate the tandem MPER repeats and stabilize each MPER module in a coiled-coil configuration. Structural predictions suggested that this modification improved the spatial organization of the tandem MPER modules while preserving the overall trimeric architecture of the immunogen (Figure 7a).

The flow cytometry binding curve demonstrated higher antigen exposure relative to the preceding design stage (Figure 7b), and AUC (FLOW) values were increased accordingly. ELISA AUC values were similar between design stage 4 and design stage 5 (Figure 7c). In contrast to earlier design stages, neutralization assays performed after three immunizations showed that the stage 5 vaccine design could induce neutralizing activity against the CNE55 pseudovirus (Figure 7d).

### 3.8. Statistical Comparison Between Successive Design Stages

To quantitatively evaluate the effect of successive vaccine design modifications on antibody binding responses, ELISA AUC values measured after the third immunization (week 9, three-dose regimen) were compared between design stages using animal-level data. All comparisons were performed using the Wilcoxon rank sum test, with effect sizes estimated by Cliff’s delta.

**Figure 8:**
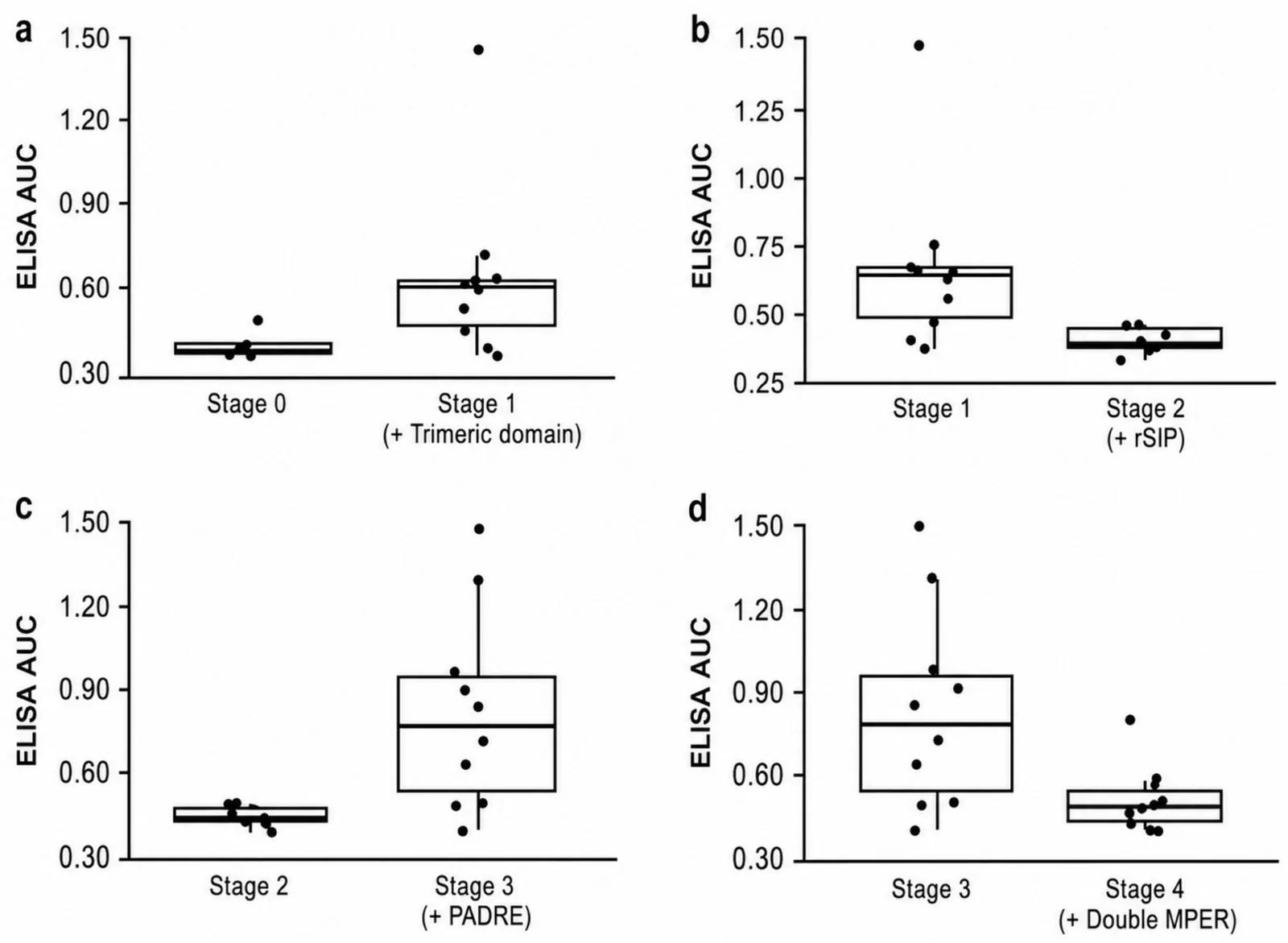
Statistical comparison of antibody binding across successive MPER vaccine design stages. Each panel compares animal-level ELISA area under the curve (AUC) values between two consecutive design stages. (**a**) Design stage 0 (MPER only baseline) versus design stage 1 (addition of heterologous trimerization domains); binding increased. (**b**) Design stage 1 versus design stage 2 (addition of rSIP); binding decreased. (**c**) Design stage 2 versus design stage 3 (addition of PADRE); binding increased. (**d**) Design stage 3 versus design stage 4 (tandem duplication of the MPER sequence without additional spacing elements); binding decreased. All four comparisons differed significantly (Wilcoxon rank-sum test): (**a**) *p* = 0.032, Cliff’s delta = −0.72 (95% CI −0.95 to −0.01); (**b**) *p* = 0.004, Cliff’s delta = −0.77 (95% CI −0.95 to −0.24); (**c**) *p* = 0.013, Cliff’s delta = −0.67 (95% CI −0.93 to −0.04); and (**d**) *p* = 0.031, Cliff’s delta = −0.58 (95% CI −0.87 to −0.01). Boxes indicate the median and interquartile range; whiskers extend to the most extreme values lying within 1.5 times the interquartile range of the box. All individual animal values are plotted, including those falling outside the whiskers. Abbreviations: MPER, membrane proximal external region; rSIP, recombinant Group B streptococcal surface immunogenic protein; PADRE, Pan DR epitope; ELISA, enzyme-linked immunosorbent assay; AUC, area under the curve.

Introduction of trimerization domains at design stage 1 resulted in a significant increase in ELISA AUC relative to the MPER-only baseline (Wilcoxon *p* = 0.032; Cliff’s delta = −0.72, 95% CI −0.95 to −0.01), consistent with improved antibody binding following stabilization of MPER within a trimeric assembly (Figure 8a).

In contrast, incorporation of rSIP at design stage 2 was associated with a significant reduction in ELISA AUC compared with design stage 1 (Wilcoxon *p* = 0.004; Cliff’s delta = −0.77, 95% CI −0.95 to −0.24) (Figure 8b), suggesting that addition of large proximal structural elements can negatively influence MPER immunogenicity despite maintained antigen exposure.

Addition of PADRE at design stage 3 reversed this effect, producing a significant increase in antibody binding responses relative to rSIP-containing candidates lacking PADRE (Wilcoxon *p* = 0.013; Cliff’s delta = −0.67, 95% CI −0.93 to −0.04) (Figure 8c). These findings support the contribution of helper T cell stimulation to enhancement of MPER-directed antibody responses within the KWC/GRB platform.

Finally, increasing MPER valency through tandem duplication of the MPER sequence at design stage 4, without incorporation of the additional spacing elements introduced in later stages, resulted in a significant reduction in ELISA AUC relative to design stage 3 (Wilcoxon *p* = 0.031; Cliff’s delta = −0.58, 95% CI −0.87 to −0.01) (Figure 8d). Together with the structural predictions described above, these findings suggested that tandem MPER duplication alone was insufficient to improve immunogenicity and that additional structural organization of duplicated MPERs would likely be required to support improved functional presentation.

### 3.9. Comparison of the Effects of Including Different Design Features

Because none of the vaccine designs evaluated in earlier stages induced detectable neutralizing activity, and because structural predictions suggested that tandem duplication of MPER alone resulted in close spatial proximity between adjacent MPER repeats and a potentially unfavorable tertiary/quaternary organization of the tandem MPER module, we introduced an additional Foldon trimerization domain between the two MPER units in design stage 5. The goal was to better separate the tandem MPER repeats and stabilize the trimeric coiled-coil conformation of each MPER.

All other vaccine components were kept constant between design stage 4 and design stage 5, allowing direct evaluation of the effect of the additional Foldon spacing and trimerization element. ELISA AUC values did not differ significantly between the two design stages (Wilcoxon *p* = 0.29), although a moderate effect size was observed (Cliff’s delta = 0.44), with confidence intervals overlapping zero. In contrast to the comparable ELISA distributions, vaccine-level flow cytometry metrics differed between the two stages. Design stage 5 exhibited higher MPER exposure, with exposure_score increasing from −2.1 to −1.8 and AUC (FLOW) increasing from 133 to 169 relative to design stage 4.

Most importantly, neutralizing activity was detected exclusively in design stage 5, following three immunizations, whereas no neutralization was detected for design stage 4 under identical experimental conditions. The design stage 5 vaccine neutralized the tier 2 pseudovirus CNE55, while the corresponding design stage 4 vaccines, lacking the additional Foldon spacing and trimerization stabilization domain, remained non-neutralizing. These findings suggest that improved spatial organization and conformational stabilization of the tandem MPER repeats were associated with the emergence of neutralizing activity in a subset of mice. Importantly, this comparison partially controls for antigen amount and immunization schedule, because design stages 4 and 5 encoded the same number of MPER copies, were administered using the identical three-dose regimen, and elicited comparable ELISA binding responses (*p* = 0.29), yet only design stage 5 induced detectable neutralization. This pattern is consistent with the interpretation that the geometry and conformational organization of the displayed MPER, rather than the amount of bound antibody alone, contributed to the emergence of neutralizing activity. We note, however, that design stage 5 also showed a modest increase in surface MPER signal (AUC (FLOW) 133 to 169), so a contribution from increased antigen display cannot be excluded, and the small group sizes limit the strength of this inference. We therefore present this as one interpretation consistent with the data rather than as a definitive mechanistic claim.

### 3.10. Comparative Statistical Analysis of Foldon- and Zipper-Based Designs

To assess the impact of trimerization stabilization on antibody binding responses, ELISA AUC values were compared between Foldon- and Zipper-containing candidates at design stages in which both architectures were evaluated. Analyses were performed using animal-level data and the Wilcoxon rank-sum test. At design stage 1, when Foldon and Zipper domains were first introduced to stabilize MPER homotrimers, ELISA AUC values were comparable between the two architectures (Wilcoxon *p* = 0.68), with only a small effect size (Cliff’s delta = 0.20). Following the addition of rSIP in design stage 2, antibody binding responses remained similar between Foldon- and Zipper-stabilized designs, with no statistically significant difference in ELISA AUC (Wilcoxon *p* = 0.84; Cliff’s delta = −0.12). A similar pattern was observed at design stage 3, which incorporated PADRE into stabilized MPER vaccines. At this stage, ELISA AUC values again did not differ significantly between designs (Wilcoxon *p* = 0.68), although the estimated effect size showed a modest directional trend favoring Foldon-based candidates (Cliff’s delta = 0.20).

**Figure 9:**
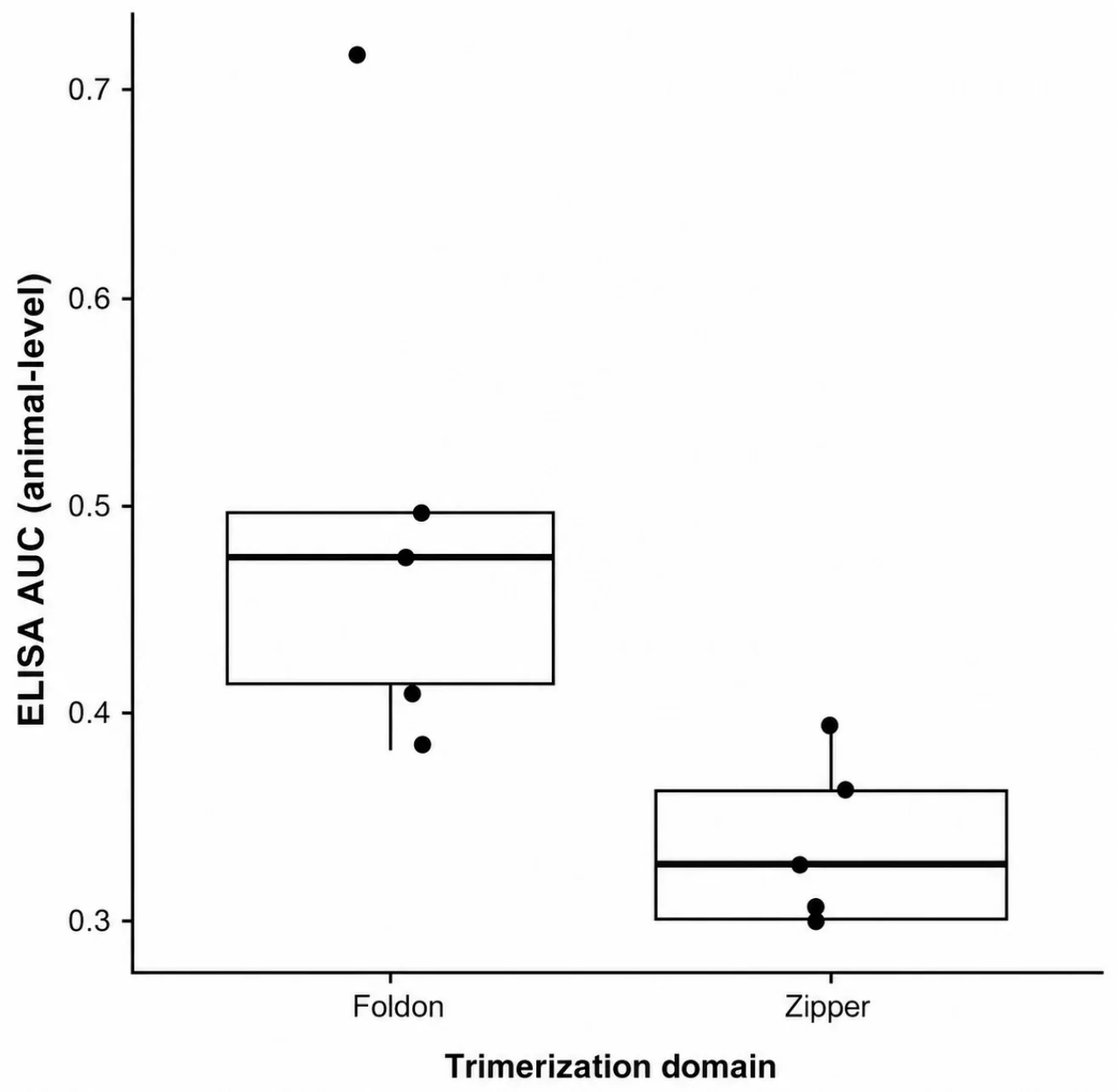
Comparison of antibody binding between designs that used either the Foldon or the Zipper domain to stabilize the MPER trimer at design stage 4. ELISA antibody binding responses are shown as animal-level area under the curve (AUC) values measured three weeks after the third immunization, isolating the effect of trimerization strategy under conditions of increased MPER valency. Foldon-based candidates showed higher ELISA AUC values than Zipper-based candidates (Wilcoxon rank-sum test, *p* = 0.021), with a large effect size favoring Foldon (Cliff’s delta = 0.92, 95% CI 0.52 to 0.99). Boxes indicate the median and interquartile range; whiskers extend to the most extreme values lying within 1.5 times the interquartile range of the box. All individual animal values are plotted, including those falling outside the whiskers. Abbreviations: MPER, membrane proximal external region; ELISA, enzyme-linked immunosorbent assay; AUC, area under the curve.

By contrast, a clear effect emerged at design stage 4, corresponding to increased MPER valency through duplication of the MPER sequence. Under these conditions, designs that incorporated Foldon elicited significantly higher ELISA AUC values than designs that used the Zipper domain to stabilize the MPER trimer (Wilcoxon *p* = 0.021), with a large effect size favoring Foldon-based vaccines (Cliff’s delta = 0.92, 95% CI 0.52–0.99) (Figure 9).

### 3.11. Vaccine-Level Analysis of Antigen Exposure and Cross-Assay Relationships

To evaluate whether increased MPER surface exposure was associated with stronger antibody binding responses, flow cytometry metrics (exposure_score and AUC (FLOW)) were compared with mean ELISA AUC values across vaccine designs. Neither exposure_score nor AUC (FLOW) showed a significant association with mean ELISA AUC (Spearman ρ ≤ 0.35, *p* ≥ 0.36; Pearson r ≤ 0.24, *p* ≥ 0.54).

These findings indicated that increased MPER surface exposure alone did not predict the magnitude of antibody binding responses measured by ELISA. Instead, the overall structural organization of the antigen appeared to play an important role in determining vaccine immunogenicity. Together with the neutralization data, these results suggested that effective MPER vaccine design depends not only on antigen exposure, but also on how MPER is spatially presented within the trimeric assembly.

### 3.12. Extended Immunization Using MPER Vaccines of Design Stage 5

**Figure 10:**
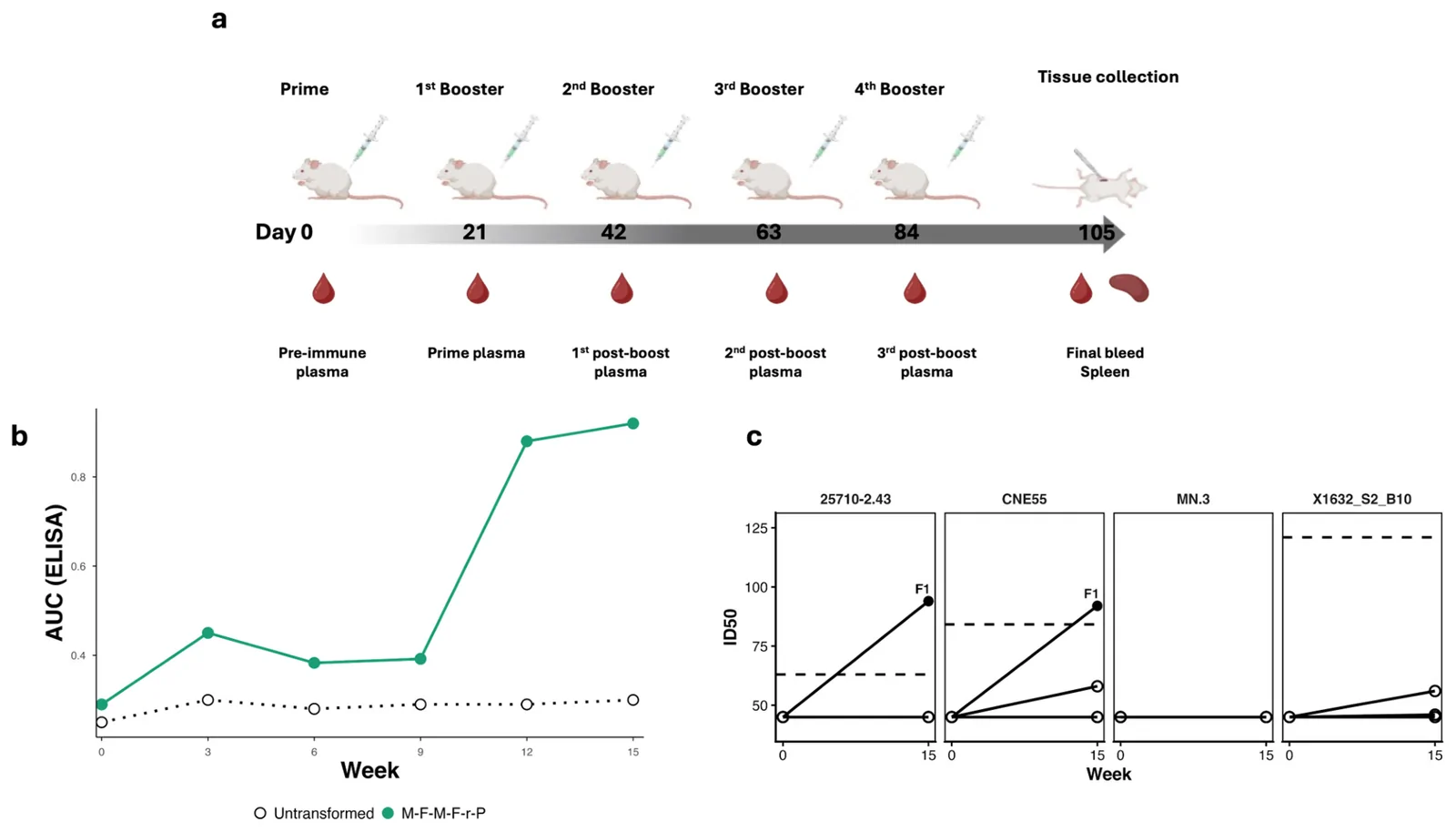
Extended immunization of the design stage 5 vaccine induces antibodies with increased binding and expanded neutralization. (**a**) Schematic of the extended immunization regimen. Mice received five intramuscular immunizations at days 0, 21, 42, 63, and 84, followed by terminal tissue collection at day 105. Plasma samples were collected prior to immunization and after each dose. (**b**) Longitudinal MPER-specific antibody binding measured by ELISA and expressed as area under the curve (AUC). The design stage 5 vaccine showed a marked increase in antibody binding following additional booster doses, whereas responses in mice immunized with untransformed bacteria remained low and stable. Filled circles represent mice immunized with the design stage 5 vaccine, and open circles represent mice immunized with formalin-inactivated untransformed bacteria. (**c**) Neutralization activity of plasma from mice immunized with the design stage 5 vaccine against a panel of HIV-1 Env pseudoviruses. After three immunizations, neutralization was detected against CNE55, and extension of immunization to five doses resulted in increased neutralization titers against CNE55 and the emergence of neutralization against an additional tier 2 virus, 25710-2.43. No neutralization was observed against MN.3 or X1632_S2_B10. Each line represents an individual animal, and dashed lines indicate virus-specific ID50 positivity cutoffs. Filled black circles connected by black lines denote neutralization-positive animals, whose plasma ID50 exceeded the virus-specific cutoff, whereas open circles connected by gray lines denote animals that remained below the cutoff. Labeled points (e.g., F1) indicate the identifiers of individual neutralization-positive animals (F, female; M, male). Abbreviations: MPER, membrane proximal external region; ELISA, enzyme-linked immunosorbent assay; AUC, area under the curve; ID50, 50% inhibitory dilution.

Following the detection of neutralizing activity for design stage 5 after three immunizations, we hypothesized that additional vaccinations would yield better neutralization responses, as had been observed for other immunogens [25], we evaluated the ability of design stage 5 vaccines to induce immune responses with an extended immunization regimen (Figure 10a). No changes to the vaccine design were introduced in these experiments.

ELISA AUC values were measured at later time points during the extended immunization regimen, revealing increased antibody binding responses over the course of five immunizations (Figure 10b). Neutralization assays performed after extended immunization showed continued neutralizing activity against CNE55 and the emergence of neutralization against an additional pseudovirus, 25710-2.43, with measurable ID50 values (Figure 10c).

### 3.13. Structural Refinement of Design Stage 5 Through Insertion of an eHIAs Spacer

Although design stage 5 induced detectable neutralizing activity and showed improved functional performance after extended immunization, inspection of the predicted trimeric structure suggested that further local optimization of MPER presentation might be possible. In particular, the MPER repeat positioned proximal to Hia appeared partially resident within the predicted Hia barrel envelope. Because this region is directly adjacent to the bacterial surface display scaffold, we reasoned that the spacing between the proximal MPER domain and Hia could influence outward projection and epitope accessibility of the MPER domain. We hypothesized that partial positioning of MPER within the Hia β-barrel envelope could reduce epitope accessibility and thereby limit antibody induction compared with designs in which MPER is projected farther beyond the Hia barrel.

**Figure 11:**
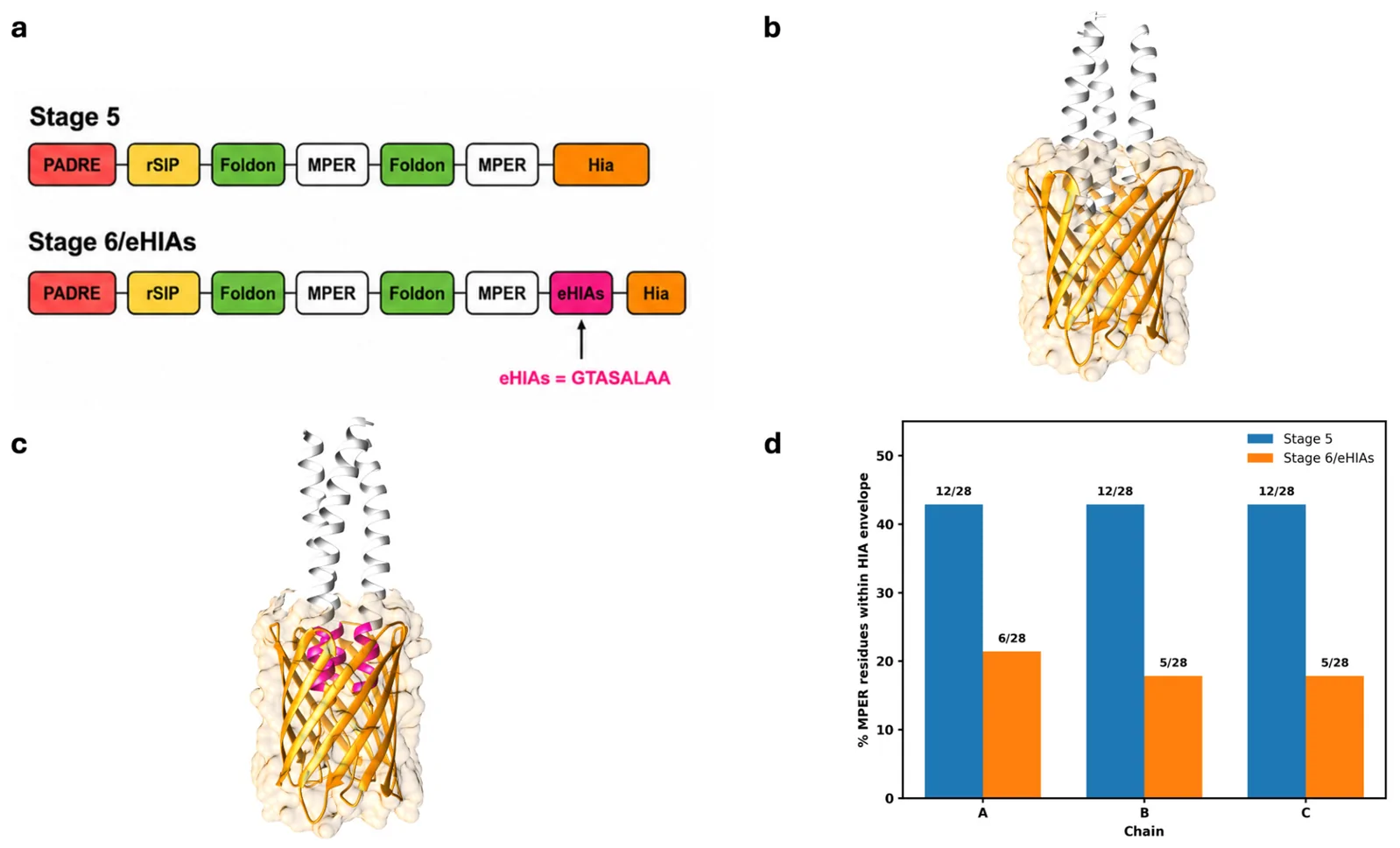
Structural refinement of design stage 5 through insertion of an eHIAs spacer. (**a**) Schematic representation of design stage 5 and design stage 6/eHIAs. Design stage 6 contains the eight amino acid eHIAs spacer sequence GTASALAA inserted between the proximal MPER repeat and Hia. PADRE is shown in red, rSIP in yellow, Foldon in green, MPER in white, eHIAs in pink, and Hia in orange. (**b**) Zoomed view of the predicted structure Hia-proximal MPER in design stage 5, showing partial positioning of the proximal MPER repeat within the predicted Hia barrel envelope. (**c**) Corresponding zoomed view of the predicted structure design stage 6/eHIAs, showing eHIAs positioned at the Hia-MPER junction and outward displacement of the proximal MPER repeat. (**d**) Quantification of proximal MPER residues positioned within the predicted Hia envelope across chains A, B, and C. Design stage 5 contained 12 of 28 MPER residues within the Hia envelope in each protomer, whereas design stage 6/eHIAs contained 6 of 28 residues in chain A and 5 of 28 residues in chains B and C. Abbreviations: MPER, membrane proximal external region; Hia, Haemophilus influenzae adhesin; rSIP, recombinant Group B streptococcal surface immunogenic protein; PADRE, Pan DR epitope.

To address this potential structural limitation, we generated an otherwise identical derivative of the design stage 5 vaccine that contained an extra eight amino acid eHIAs spacer sequence, GTASALAA, inserted between the proximal MPER repeat and the Hia β-barrel/translocator domain. This candidate was defined as design stage 6 (Figure 11a). A structural comparison of the Hia-proximal region suggested that, in design stage 5, a portion of the proximal MPER repeat remained positioned within the predicted Hia barrel envelope (Figure 11b). In contrast, insertion of the 8 amino acid eHIAs shifted the membrane-proximal MPER repeat outward relative to the Hia barrel (Figure 11c).

To quantify this local structural change across the trimeric assembly, we performed an Hia barrel envelope analysis. The Hia region was used to define the predicted axial and radial envelope of the barrel, and each residue of the proximal MPER repeat was classified relative to this envelope in chains A, B, and C. In design stage 5, 12 of 28 MPER residues were positioned within the predicted Hia envelope in each protomer. In design stage 6, this value was reduced to 6 of 28 residues in chain A and 5 of 28 residues in chains B and C (Figure 11d). Consistent with this reduction, the mean axial position of the proximal MPER repeat shifted from 26.4 Å in design stage 5 to 37.2 Å in design stage 6, corresponding to an outward displacement of approximately 10.7 Å along the Hia barrel axis.

Together, these analyses indicated that eHIAs did not completely eliminate Hia-proximal inclusion of MPER but substantially reduced the fraction of the proximal MPER repeat positioned within the predicted Hia barrel envelope. These findings supported design stage 6 as a rational architectural refinement intended to improve outward presentation of the MPER module while preserving the design logic established in stage 5.

### 3.14. Design Stage 6 Enhances MPER Exposure, Increases Antibody Binding, and Is Associated with a Distinct Neutralization Profile

**Figure 12:**
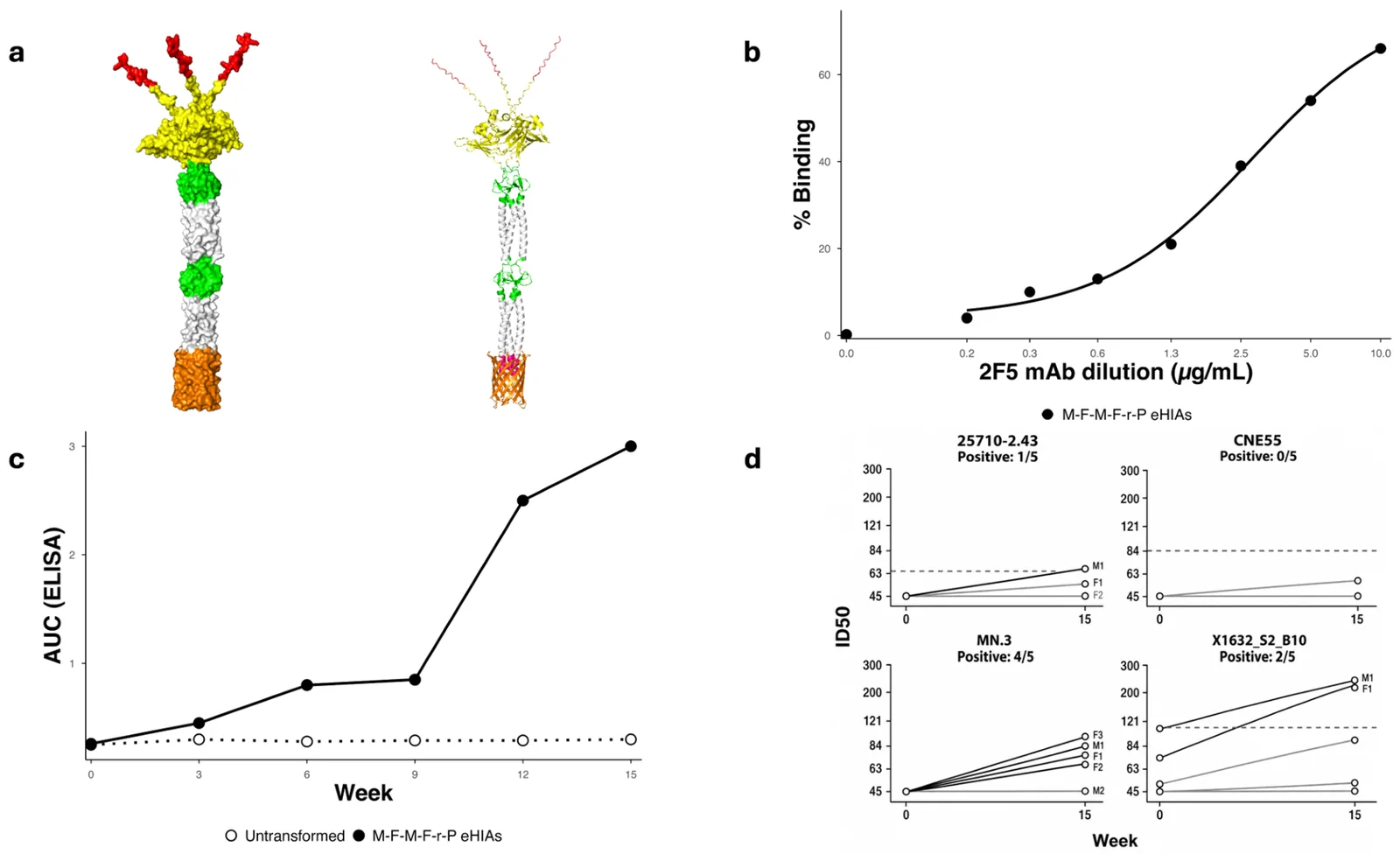
Design stage 6/eHIAs enhances MPER exposure, increases antibody binding, and is associated with a distinct neutralization profile. (**a**) Predicted three-dimensional structure of the design stage 6/eHIAs vaccine, M-F-M-F-r-P eHIAs. The design is shown as both a surface/cartoon representation and a cartoon model to visualize the Hia barrel, repeated MPER helices, and the eHIAs spacer. PADRE is shown in red, rSIP in yellow, Foldon in green, MPER in white, eHIAs in pink, and Hia in orange. (**b**) Flow cytometry analysis of MPER surface exposure measured using the broadly neutralizing monoclonal antibody 2F5. Formalin-inactivated bacteria expressing the design stage 6/eHIAs vaccine were incubated with increasing concentrations of 2F5, and binding is reported as percent positive cells. (**c**) Longitudinal MPER-specific antibody binding measured by ELISA and expressed as area under the curve (AUC) following the extended immunization regimen. Filled circles represent mice immunized with M-F-M-F-r-P eHIAs, and open circles represent mice immunized with formalin-inactivated untransformed bacteria. (**d**) Neutralization activity of plasma from mice immunized with the design stage 6/eHIAs vaccine against 25710-2.43, CNE55, MN.3, and X1632_S2_B10 HIV-1 Env pseudoviruses. Each line represents an individual animal, and dashed horizontal lines indicate virus-specific ID50 positivity cutoffs. Filled black circles connected by black lines denote neutralization-positive animals, whose plasma ID50 exceeded the virus-specific cutoff; open circles connected by gray lines denote animals that remained below the cutoff. Labeled points indicate the identifiers of individual neutralization-positive animals (F, female; M, male). Positivity was detected in 1 of 5 animals against 25710-2.43, 0 of 5 against CNE55, 4 of 5 against MN.3, and 2 of 5 against X1632_S2_B10. Abbreviations: MPER, membrane-proximal external region; Hia, Haemophilus influenzae adhesin; rSIP, recombinant Group B streptococcal surface immunogenic protein; PADRE, Pan-DR epitope; ELISA, enzyme-linked immunosorbent assay; AUC, area under the curve; ID50, 50% inhibitory dilution; mAb, monoclonal antibody.

Based on the structural refinement described above, we next evaluated how insertion of the eHIAs spacer affected experimental performance. The design stage 6/eHIAs vaccine, M-F-M-F-r-P eHIAs, preserved the overall modular organization of design stage 5 while incorporating the eight amino acid eHIAs spacer between the proximal MPER repeat and Hia (Figure 12a). To visualize this design, the predicted structure is shown both as a surface/cartoon representation and as a cartoon model, allowing visualization of the Hia barrel, the repeated MPER helices, and the eHIAs region.

Flow cytometry analysis using the broadly neutralizing monoclonal antibody 2F5 showed strong MPER surface accessibility for the design stage 6/eHIAs vaccine (Figure 12b). Binding increased in a concentration-dependent manner across the 2F5 dilution range, indicating that the MPER epitope recognized by 2F5 remained accessible after insertion of the eHIAs spacer. These findings were consistent with the structural prediction that eHIAs shifted the Hia-proximal MPER repeat outward rather than disrupting the MPER-containing trimeric module.

We next evaluated MPER-specific antibody binding responses following the extended immunization regimen. ELISA AUC values increased progressively over time in mice immunized with the design stage 6/eHIAs vaccine, with the strongest responses observed after later booster doses (Figure 12c). In contrast, mice immunized with formalin-inactivated untransformed bacteria remained near baseline throughout the experiment. Thus, the eHIAs-containing vaccine induced a robust longitudinal MPER-specific antibody response under the five-dose regimen.

Finally, we evaluated the neutralizing activity of plasma from mice immunized with the design stage 6/eHIAs vaccine against a panel of HIV-1 Env pseudoviruses. Neutralization was virus-dependent and differed from the profile observed previously for design stage 5. Using virus-specific positivity cutoffs, neutralizing activity was detected in 1 of 5 animals against 25710-2.43, 0 of 5 animals against CNE55, 4 of 5 animals against MN.3, and 2 of 5 animals against X1632_S2_B10 (Figure 12d). Therefore, eHIAs insertion did not simply increase neutralization uniformly across the panel but instead was associated with a different, virus-dependent neutralization profile, with detectable activity against multiple pseudoviruses. Because only a subset of animals responded, this pattern is descriptive and does not by itself establish that eHIAs altered antibody specificity.

Together, these results indicate that the design stage 6 eHIAs vaccine maintained strong MPER surface exposure, induced robust MPER-specific antibody binding after extended boosting, and generated a distinct neutralization profile across the tested HIV-1 pseudovirus panel. These findings support eHIAs-mediated Hia-proximal spacing as a tunable architectural parameter for improving MPER presentation in the KWC/GRB platform.

## 4. Discussion

The trimeric coiled-coil domains of envelope proteins from viruses with class I viral fusion proteins have been key targets for the development of vaccines intended to induce broadly neutralizing and/or variant-resistant immune responses. Although the existence of broadly neutralizing monoclonal antibodies targeting these regions demonstrates that such effective antibody responses can, in principle, be generated, the development of vaccines that reliably induce them has remained challenging. The HIV-1 gp41 membrane-proximal external region (MPER) remains an attractive but extremely challenging target within this broader class of potential immunogens. MPER is highly conserved and is recognized by several broadly neutralizing antibodies, but its native antigenic presentation depends on several structural features that are difficult to reproduce in a vaccine immunogen: MPER must exist in the native coiled-coil conformation, and ideally the MPER immunogen would be expressed in close proximity to the viral membrane, since some anti-MPER broadly neutralizing monoclonal antibodies interact not only with MPER itself, but also with the viral envelope lipid bilayer through long hydrophobic loops [6,8,13,14,68]. These features make MPER-directed immunogenicity highly dependent on how the epitope is displayed [69,70,71,72]. In this study, we used the KWC/GRB platform to express native-sequence MPER immunogens on the bacterial surface using a trimeric autotransporter in immediate proximity to the lipid bilayer of the bacterial outer membrane. We note that positioning MPER adjacent to the bacterial outer membrane was a biologically motivated design rationale rather than an experimentally isolated variable; accordingly, membrane-proximal display is best regarded as a plausible contributing factor rather than a demonstrated determinant of the neutralizing responses observed here. In our immunogen design campaign, we progressively modified immunogen architecture through defined design stages. Building on prior KWC/GRB studies with viral fusion peptide and scaffolded MPER immunogens [33,34,35], our results demonstrate that successful MPER presentation depends not only on antigen exposure, but also on the spatial organization of MPER relative to trimerization domains, immunomodulatory elements, tandem antigen repeats, and the Hia display scaffold.

Across the sequential development of the KWC/GRB platform, prior studies established several principles that informed the present work. The initial coronavirus fusion peptide study demonstrated that genome-reduced bacteria could be used as a killed whole-cell chassis for surface display of conserved viral fusion epitopes and could induce protective immunity in a relevant animal model. Subsequent optimization using class I viral fusion peptides showed that surface exposure is a key determinant of humoral immunogenicity and that antigen multimerization, linker design, and incorporation of immunomodulatory elements can substantially increase antigen-specific antibody responses. The scaffolded MPER study then showed that structural stabilization of a difficult membrane-proximal epitope could enable the induction of neutralizing activity in the KWC/GRB platform. Together, those studies suggested that successful KWC/GRB vaccine design requires coordinated optimization of antigen exposure, structural constraint, modular immunostimulation, and epitope accessibility. The present study extends these principles to native-sequence MPER displayed as a trimeric coiled-coil immunogen and identifies additional requirements related to trimeric stabilization, tandem antigen spacing, and antigen-scaffold geometry.

The strategy of presenting MPER in a trimeric configuration has been explored previously, although in structural contexts distinct from the one used here. Soluble recombinant gp41-based immunogens designed to stabilize MPER within a trimeric coiled-coil, including constructs in which MPER and gp41 heptad-repeat regions were constrained by heterologous coiled-coil domains [73,74], were efficiently recognized by broadly neutralizing antibodies and elicited MPER-directed antibody responses in rabbits, but induced little or no HIV-1 neutralizing activity. Similarly, repetitive display of MPER on self-assembling protein nanoparticles built from pentameric and trimeric coiled-coil domains raised high MPER-specific titers without added adjuvant, yet did not induce detectable neutralization [75]. Trimeric gp41 stabilized in a prehairpin-like conformation and displayed on bacteriophage T4 capsids has also been engineered to expose the 2F5 and 4E10 epitopes [28]. Together, these studies indicate that trimerization of MPER, although important for approximating the native quaternary organization of the epitope, has generally been insufficient on its own to elicit neutralizing antibodies. To our knowledge, the present study is the first to display native-sequence MPER as a trimeric coiled-coil through a bacterial trimeric autotransporter on the surface of a killed whole-cell vaccine, positioning the epitope adjacent to a lipid bilayer and, in the most advanced designs, co-expressing it in a single genetically encoded construct with immunomodulatory elements. This configuration differs from prior soluble, nanoparticle, and phage-displayed trimeric MPER immunogens in both display geometry and membrane-proximal context and provides a distinct immunogen-display context associated with the detectable, virus-dependent neutralizing activity reported here.

A major conclusion from this work is that structural reinforcement of MPER within a trimeric coiled-coil context improves immunogen performance relative to an MPER-only baseline. Incorporation of heterologous trimerization domains increased 2F5 binding and enhanced MPER-specific antibody responses, supporting the idea that native-sequence MPER benefits from additional stabilization when displayed on the bacterial surface. This interpretation is consistent with structural and vaccine-design studies showing that Env and gp41 immunogenicity are strongly influenced by trimeric organization and conformational stabilization [27,28,29,30,76,77,78,79,80]. This effect became particularly important as immunogen complexity increased. Simple tandem duplication of MPER increased antigen valency but did not proportionally improve antibody binding or induce neutralization, indicating that repeated antigen units require appropriate spatial separation and stabilization. Structural predictions of simple duplicated, stacked MPERs suggested that, without an intervening trimerization domain such as Foldon, the MPER repeats might not maintain favorable tertiary and quaternary organization. Thus, MPER valency alone was insufficient; the repeated MPER modules had to be organized and stabilized in a geometry compatible with epitope accessibility and functional antibody induction.

The comparison between Foldon- and Zipper-based designs further emphasizes that trimerization domains are not merely passive oligomerization tools. Although both domains supported MPER trimerization in early design stages, Foldon-based candidates performed better than Zipper-based candidates when MPER valency was increased. Foldon and zipper motifs have well-established trimerization properties, but their different structural characteristics can impose different geometric constraints on fused immunogens [45,46]. In this setting, Foldon may better preserve the spacing, rigidity, or relative orientation of tandem MPER units, thereby supporting a more favorable trimeric display. These findings highlight the importance of treating trimerization domains as architectural determinants of immunogen presentation rather than interchangeable structural accessories. Although Foldon outperformed Zipper in the designs tested here, these results do not imply that Foldon is the optimal trimerization or spacing domain. Within the scope of the present data, the advantage of Foldon over Zipper was observed specifically in tandem, dual MPER designs, whereas the two domains performed comparably with a single MPER repeat. We therefore interpret Foldon as preferable when multiple MPER repeats must be spatially organized, and we present this as a working hypothesis rather than a general design rule, since we did not systematically compare the rigidity, spacing, or flexibility of the two scaffolds. Rather, they suggest that trimerization domains can be engineered as functional spacing elements, and that future designs may benefit from alternative domains that better separate repeated MPER units, preserve coiled-coil organization, and promote productive B-cell receptor engagement, while ideally having minimal immunogenicity themselves.

The effects of immunomodulatory elements were more context-dependent. PADRE incorporation was associated with increased antibody binding responses, consistent with the expected benefit of enhanced helper T cell stimulation [47,48,49]. In contrast, rSIP-containing candidates showed reduced ELISA AUC values in some comparisons despite maintaining measurable MPER exposure by flow cytometry. This result does not necessarily indicate that rSIP is detrimental. rSIP has been described as an immunomodulatory protein with TLR2 and TLR4 agonist activity and potential adjuvant properties [50,51]. However, in the context of these MPER candidates, large accessory domains may also influence the magnitude, orientation, or specificity of the antibody response by changing the geometry of the displayed antigen. This may be especially relevant for MPER, because several MPER-directed broadly neutralizing antibodies recognize membrane-proximal or structurally constrained epitopes with specific angles of approach and, in some cases, membrane interactions [5,13,14,69,81,82,83,84]. Therefore, a reduction in total antibody binding does not necessarily imply poorer functional quality. Immunomodulatory sequences may help shape the immune response, but their effect appears to depend strongly on the structural context in which they are presented.

A recurring theme in this dataset is the dissociation between MPER accessibility, antibody magnitude, and neutralizing activity. Several vaccines displayed measurable 2F5 binding by flow cytometry and induced MPER-specific antibodies by ELISA yet failed to induce detectable neutralization after three immunizations. Conversely, design stage 5 induced neutralizing activity despite not being the strongest vaccine by every binding metric. These findings indicate that 2F5 binding and ELISA AUC are useful but incomplete predictors of functional immunogenicity, and that in vivo evaluation remains essential because current in vitro and in silico metrics do not yet fully predict functional neutralization. Flow cytometry reports whether a known neutralizing epitope is accessible on the bacterial surface, and ELISA measures the magnitude of MPER-specific antibody binding, but neither assay fully captures whether the induced antibodies recognize MPER in a geometry compatible with viral neutralization. In this context, both binding assays used here interrogate primarily the linear MPER determinant. The monoclonal antibody 2F5 recognizes a continuous (linear) epitope, so flow cytometry reports the surface availability of this linear determinant, and the ELISA capture antigen was likewise a linear MPER peptide (residues 656–683). Consequently, neither assay would detect with high efficiency antibodies directed against conformational, quaternary, or membrane-dependent MPER epitopes—precisely the specificities most often associated with MPER-directed neutralization. ELISA AUC therefore likely underestimates the functionally relevant anti-MPER response, providing an additional, non-mutually exclusive explanation for the dissociation between binding magnitude and neutralizing activity observed across our designs. Similar distinctions between antibody binding, epitope presentation, and neutralization have been emphasized in MPER and Env immunogen studies [69,70,84,85,86,87,88,89].

Design stage 5 represented the first functional breakthrough in this series. This candidate incorporated duplicated MPER repeats separated by an additional Foldon domain, thereby stabilizing each MPER module within a more organized trimeric arrangement. After three immunizations, design stage 5 induced neutralizing activity against CNE55, whereas earlier design stages remained non-neutralizing. Extending the immunization regimen increased MPER-specific antibody binding and expanded detectable neutralization to an additional pseudovirus, 25710-2.43. Because detectable neutralization first emerged in design stage 5 already after three immunizations—the same regimen under which design stages 0–4 remained non-neutralizing—the extended regimen appears to have expanded and strengthened, rather than created this response. Nevertheless, we cannot fully separate the contribution of additional boosting to antibody quantity from effects on antibody quality, and this alternative is acknowledged in the study limitations. These results show that native-sequence MPER, when organized as a repeated trimeric coiled-coil on the KWC/GRB surface, can prime antibody responses with measurable antiviral function. They also suggest that repeated antigen exposure can amplify or mature functional responses that may initially be weak or restricted, consistent with broader HIV vaccine studies showing that sequential or repeated immunization can promote antibody maturation and functional activity [25,80,90,91,92].

The design stage 6 experiment extends this conclusion by showing that detailed antigen-scaffold geometry remains important even after tandem MPER repeats have been separated and stabilized by an intervening trimerization domain. Structural analysis of design stage 5 indicated that the MPER repeat proximal to Hia remained partially located within the predicted Hia barrel envelope. This observation motivated insertion of the short eHIAs spacer, GTASALAA, between the proximal MPER repeat and the Hia β-barrel/translocator domain. Barrel-envelope analysis of the predicted models suggested that eHIAs reduced the predicted fraction of proximal MPER residues positioned within the Hia envelope from 12 of 28 residues per protomer in design stage 5 to 5–6 of 28 residues per protomer in design stage 6, with the mean axial position of the proximal MPER repeat predicted to shift outward by approximately 10.7 Å. Because these models carried low global and interface confidence (pTM ≈ 0.30–0.34, ipTM ≈ 0.31–0.33 for these designs), these values are interpreted as relative, model-based design metrics rather than as accurate structural measurements. These structural analyses relied on AlphaFold-based predictions and visualization/analysis of predicted models, approaches that are valuable for immunogen design but should be interpreted cautiously in the absence of experimentally solved structures [52,53,54,55,56]. Thus, these predicted structural changes do not establish causality, but they provide a rational structural basis for why a minimal Hia-proximal spacer could alter MPER exposure and downstream immunogenicity without changing the MPER antigen sequence itself. These findings support further optimization of Hia-proximal spacing as a design variable for improving MPER presentation and functional antibody induction.

Experimentally, the design stage 6/eHIAs vaccine showed strong 2F5 binding, robust longitudinal MPER-specific ELISA responses, and a distinct neutralization profile. Importantly, eHIAs did not simply improve neutralization uniformly across all pseudoviruses. Instead, it was associated with a distinct functional pattern of the antibody response, with detectable activity against 25710-2.43, MN.3, and X1632_S2_B10, but not CNE55 under the conditions tested. This virus-dependent pattern is informative because it raises the possibility that small changes in Hia-proximal spacing may influence the antibody populations elicited by the vaccine. However, because neutralization was detected in only a small number of animals and the stage 5 and stage 6 profiles were compared across different responders, these data are descriptive and do not establish that eHIAs altered antibody specificity. With this caveat, eHIAs is best interpreted not as a universal enhancer of neutralization, but as an architectural refinement that is associated with a shifted, virus-dependent neutralization profile, a hypothesis that will require larger, appropriately powered studies to test. Neutralization was assessed using established HIV pseudovirus neutralization approaches and virus-specific positivity cutoffs [59,60]. Although outside the scope of the present study, the virus-dependent neutralization patterns observed here raise the possibility that different trimeric MPER architectures may preferentially elicit partially distinct functional antibody populations. Future multivalent formulations combining complementary MPER immunogen architectures could therefore be evaluated as a strategy to broaden the neutralization profile.

Together, the design stage 5 and 6 findings support a working threshold-and-geometry model for MPER vaccine design in the KWC/GRB platform. Trimeric stabilization greatly improves the baseline organization of MPER, PADRE can enhance antibody binding responses, and Foldon-mediated separation of tandem MPER repeats can enable the emergence of neutralizing activity. However, even after these improvements, local junctional spacing between the antigen and the autotransporter scaffold remains a determinant of presentation. This interpretation is consistent with the biology of trimeric autotransporters and Hia, where trimerization, translocator architecture, and passenger-domain organization can influence surface display and stability [36,37,38,39,40,41,42,43,44]. The experiments with the eHIAs show that an eight amino acid spacer can substantially reduce Hia-proximal MPER inclusion within the predicted barrel envelope and is associated with altered functional antibody output. Thus, effective MPER display requires both appropriate global architecture and optimized local antigen-scaffold spacing. Future studies should determine whether systematic modulation of spacer length, composition, and rigidity can further improve MPER presentation and functional antibody induction.

This interpretation also helps explain why simple correlations between flow cytometry metrics and ELISA responses were weak. MPER exposure is necessary, but not sufficient, for functional immunogenicity. A vaccine can expose MPER in a way that allows binding by a monoclonal antibody or detection in ELISA yet still fail to induce antibodies with the appropriate specificity, orientation, or maturation trajectory for viral neutralization. Conversely, a vaccine with only modest differences in average binding metrics may present key epitopes in a more productive geometry. This distinction is particularly important for MPER, where neutralization depends on antibodies that recognize a structurally constrained, membrane-proximal epitope in a highly specific context [5,6,13,69,82,83,84]. Thus, the goal is not simply to maximize antigen display, but to engineer antigen presentation in a way that couples epitope accessibility, structural constraint and stabilization, and immunostimulatory context to favor functional antibody responses.

The present study has several strengths. First, it used an iterative design strategy in which individual architectural variables were evaluated while maintaining a common antigenic target and platform, extending principles previously developed with multimerized KWC/GRB immunogens displayed through the monomeric AIDA-I autotransporter [33,34,35]. Second, the study integrated contemporary immunogen-design principles with structural prediction, monoclonal antibody binding, ELISA, and neutralization assays, allowing each design stage to be evaluated at multiple complementary levels. Third, the progression from design stage 5 to design stage 6 illustrates how structural observations can guide minimal sequence modifications that are then tested experimentally. This design-build-test-learn workflow is a major advantage of the KWC/GRB platform and is consistent with prior use of the platform for rapid engineering of bacterial surface-displayed immunogens [33,34,35].

Although the vaccine designs evaluated in this study did not induce highly potent or broadly neutralizing responses in all animals, they induced detectable neutralization in subsets of mice using KWC/GRB vaccines without additional, external adjuvants. In previous studies, the induction of significant neutralization required not only priming with isolated MPER immunogens, but also subsequent challenge infection with live virus [17,18,19,20]. By contrast, the stage 5 and 6 designs elicited detectable neutralization in a subset of mice through immunization alone, without live-virus challenge or external adjuvant. We interpret this as a proof-of-concept that native-sequence MPER displayed on the KWC/GRB platform can prime functionally active antibody responses, rather than as evidence of a broadly protective or deployable HIV-1 vaccine. The neutralizing responses were modest, virus-dependent, restricted to a subset of animals, and in several cases close to the positivity threshold, and no broadly neutralizing activity was observed. The current findings provide a starting point for further optimization of trimeric Hia-displayed MPER immunogens. The use of quasi-outbred HET3 mice is also relevant because this model introduces greater host genetic diversity than standard inbred mouse strains, a potentially stricter evaluation criterion, although further studies will be required to determine how these responses translate across species.

This study has several limitations. Structural interpretations relied on low-confidence predicted models rather than experimentally solved structures [52,53], and independent structural and biochemical validation (cryo-electron microscopy, X-ray crystallography, cross-linking mass spectrometry, Western blotting, or blue-native PAGE) was not performed to confirm expression, trimer assembly, or MPER extension beyond the membrane; because all constructs shared the same expression backbone, differences in 2F5 surface binding may nonetheless reflect accessibility, display efficiency, or expression level, which we could not fully separate. Immunological characterization was limited to MPER-specific binding (ELISA and flow cytometry) and neutralization; because 2F5 and the ELISA peptide report a linear epitope, conformational and membrane-dependent responses may be underestimated, and we did not use 4E10 or 10E8 or perform epitope mapping, isotyping, affinity, or B cell analyses. The study did not include a dedicated series of MPER-containing constructs in which the presence, position, or spacing of the immunomodulatory elements was varied systematically, nor an equal-dose soluble MPER trimer; consequently, the independent contributions of rSIP and PADRE, and of antigen dose, trimer structure, and membrane microenvironment, could not be separated, and membrane-proximal display is best regarded as a plausible contributing factor rather than a demonstrated determinant. Finally, small group sizes (five animals per group) limit statistical power and render comparisons and Section 3.11 correlation exploratory rather than confirmatory; neutralization was virus-dependent and should not be interpreted as broad, and the mouse model will require further work to establish whether these responses can be strengthened, broadened, and translated across species.

Future work should pursue two complementary directions. One is continued engineering of the trimeric KWC/GRB platform, including systematic modulation of MPER-Hia spacing, alternative trimerization domains, lower-immunogenicity structural elements, evaluation of additional immunomodulatory sequences, and strategies to increase MPER valency without inducing collapse of the MPER coiled-coil trimeric structure or steric occlusion. The second is development of heterologous immunization regimens combining trimeric MPER KWC/GRB vaccines with scaffolded MPER immunogens. Scaffolded MPER designs present the epitope in a structurally constrained but distinct context, whereas the trimeric KWC/GRB vaccines present native MPER as a repeated coiled-coil array adjacent to a bacterial surface scaffold. Prior MPER epitope-scaffold studies and scaffold-based immunofocusing approaches support the rationale for combining distinct MPER presentation formats [35,71,85,93,94]. Combining these approaches may improve antibody magnitude, diversify the responding B cell repertoire, and promote more consistent functional maturation. Additional strategies could include co-administration of external adjuvants, since the present study used only the immunomodulatory elements encoded within the expressed MPER immunogen and the intrinsic adjuvanticity of the bacterial chassis. Engineering improved bacterial chassis strains may provide an additional route to tune reactogenicity, antigen visibility, and immunogenicity.

In summary, this study demonstrates that native-sequence MPER can be engineered within the KWC/GRB platform to induce MPER-specific antibodies with detectable neutralizing activity. The data identify several design principles: MPER benefits from trimeric stabilization, increased valency requires controlled spatial organization, antibody binding magnitude alone does not predict neutralization, extended boosting can enhance functional activity, and Hia-proximal spacing is a tunable determinant of MPER display. The eHIAs spacer experiment is particularly important because it shows that a minimal local architectural change can substantially alter predicted MPER presentation and is associated with a shifted functional antibody response, although the small number of responding animals means this pattern is descriptive rather than a demonstrated change in antibody specificity. As a proof-of-concept, these findings inform the rational development of MPER-targeted KWC/GRB vaccines and provide a framework for optimizing bacterial surface-displayed immunogens against structurally constrained viral epitopes, while recognizing that the neutralizing responses observed here were modest, virus-dependent, and not broadly neutralizing.

## 5. Conclusions

This study demonstrates that the KWC/GRB vaccine platform can display native-sequence HIV-1 MPER immunogens in a trimeric coiled-coil configuration and induce MPER-specific antibodies with detectable, virus-dependent neutralizing activity. Through iterative immunogen engineering, we progressed from designs that induced binding antibodies without neutralization to architectures capable of generating functional anti-HIV activity. The results identify several key design principles: trimeric stabilization improves MPER presentation, increased MPER valency requires controlled spacing and stabilization, antibody binding magnitude alone does not predict neutralization, extended boosting can enhance functional responses, and Hia-proximal spacing is a tunable determinant of MPER display.

The identification of design stage 5 established that duplicated MPER repeats separated by Foldon can induce neutralization after three immunizations and expand functional activity after extended boosting. Further refinement with the design stage 6/eHIAs vaccine showed that a minimal spacer at the Hia-proximal junction can reduce predicted MPER inclusion within the Hia barrel envelope and was associated with a shifted, virus-dependent neutralization profile across the pseudovirus panel. Although the responses observed here do not yet represent broad neutralization, these findings provide a rational framework for further optimization of MPER-directed KWC/GRB vaccines.

The primary goal of developing a safe and effective HIV vaccine remains the prevention of new infections. Long-acting antiretroviral therapy and long-acting PrEP represent major advances in HIV treatment and prevention, but they do not eliminate the need for safe, durable, scalable, and affordable vaccines [95]. Drug-based prevention strategies require sustained healthcare infrastructure, repeated clinical access, reliable supply chains, and long-term implementation capacity, which can be difficult to maintain in under-resourced settings [96]. In this context, the KWC/GRB platform has potential practical advantages because it is rapidly adaptable, potentially compatible with low-cost manufacturing, and conceptually aligned with existing killed whole-cell vaccine production approaches. If further optimized to induce broader and more potent neutralization, a KWC/GRB MPER vaccine could contribute to HIV prevention strategies, particularly in regions where access to long-term biomedical interventions remains constrained.

More broadly, the design principles developed in this study may inform KWC/GRB vaccine engineering against other viruses with class I fusion proteins, including pathogens for which conserved membrane-proximal stem or stalk regions are attractive but difficult vaccine targets. Because the present study evaluated only HIV-1 MPER, extension of this approach to other targets, such as the influenza hemagglutinin or coronavirus spike stem regions, remains a hypothesis to be tested in future work. These results should be interpreted as proof-of-concept: the neutralizing responses were modest, virus-dependent, observed only in subsets of animals, and did not constitute broadly neutralizing activity.

## Figures and Tables

**Table 1:** Vaccine components evaluated in this study, indicating the presence or absence of trimerization domains, immunomodulatory elements, MPER valency modifications, and spacing elements for each design feature.

Component	Name	Abbreviation	Function	Ref.
Antigen	Membrane-Proximal External Region	MPER	HIV target	[61]
Immunomodulators	Pan DR Epitope	PADRE	Universal CD4^+^ T-helper epitope included to support helper T-cell responses.	[34,35,48,49]
	Grp B Strep Recombinant Surface Immunogenic Protein	rSIP	Immunomodulatory protein reported to activate TLR2/TLR4 signaling; included as a candidate intrinsic adjuvant module.	[50]
Trimeric Domains	GCN4 Isoleucine-Zipper Trimer	Zipper	Stabilizes the MPER homotrimer coiled-coil configuration.	[45]
	T4 Fibritin Protein	Foldon	Stabilizes the MPER homotrimer coiled-coil configuration.	[46]
Extra HIA sequence	Extended HIA sequence	eHIAs	Modulates MPER spacing and accessibility relative to the bacterial outer membrane	[43,44]

**Table 2:** Composition of all vaccine constructs evaluated in this study.

Short Name	Composition (N → C)
M	MPER
M-F	Foldon + MPER
M-Z	Zipper + MPER
M-F-r	rSIP + Foldon + MPER
M-Z-r	rSIP + Zipper + MPER
M-F-r-P	PADRE + rSIP + Foldon + MPER
M-Z-r-P	PADRE + rSIP + Zipper + MPER
M-M-F-r-P	PADRE + rSIP + Foldon + MPER + MPER
M-M-Z-r-P	PADRE + rSIP + Zipper + MPER + MPER
M-F-M-F-r-P	PADRE + rSIP + Foldon + MPER + Foldon + MPER
M-F-M-F-r-P eHIAs	PADRE + rSIP + Foldon + MPER + Foldon + MPER + eHIAs

Note: Compositions are shown from the N-terminus to the C-terminus of the expressed immunogen sequence.

**Table 3:** Summary of experimental readouts for all vaccine constructs, including antigen exposure and antibody-binding metrics and pseudovirus neutralization results.

Vaccine ID	Design Stage	Key Design Features	Exposure_Score	AUC (FLOW)	Neutralized Pseudoviruses
M	0	MPER only	−1.4	62	None
M-F	1	Trimerization domain Foldon	−1.2	80	None
M-Z	1	Trimerization domain Zipper	−0.7	87	None
M-F-r	2	Trimerization domain Foldon + rSIP	−0.3	235	None
M-Z-r	2	Trimerization domain Zipper + rSIP	−2.0	67	None
M-F-r-P	3	Trimerization domain Foldon + rSIP + PADRE	−0.9	172	None
M-Z-r-P	3	Trimerization domain Zipper + rSIP + PADRE	−1.5	102	None
M-M-F-r-P	4	Trimerization domain Foldon + rSIP + PADRE + MPER × 2	−2.1	133	None
M-M-Z-r-P	4	Trimerization domain Zipper + rSIP + PADRE + MPER × 2	−1.3	67	None
M-F-M-F-r-P	5	Tandem MPER repeats separated by an additional Foldon domain	−1.8	169	CNE55 (subset of animals)
M-F-M-F-r-P eHIAs	6	Tandem MPER repeats separated by an additional Foldon domain and extended Hia spacer	−0.3	470	MN.3, X1632_S2_B10, 25710-2.43 (subset of animals)

Note: Exposure_score corresponds to −log_10_-transformed EC_50_ values derived from 2F5 binding curves measured by flow cytometry. AUC (FLOW) represents the integrated fluorescence signal across the antibody dilution series.

## Data Availability

The processed datasets and the annotated R analysis script supporting the findings of this study are provided in the Supplementary Materials (File S1 and File S2). Raw per-animal, per-dilution ELISA OD450 values and per-chain barrel-envelope structural measurements are available from the corresponding author upon reasonable request.

## Supplementary Materials

The following supporting information can be downloaded at: https://www.mdpi.com/article/10.3390/v18080890/s1. Table S1 (AlphaFold2-Multimer model confidence metrics: pLDDT ranges, pTM, and ipTM for all designs); Table S1: AlphaFold2-Multimer model confidence metrics: pLDDT ranges, pTM, and ipTM for all designs; File S1: R Analysis Script; File S2: Processed Datasets.

## Abbreviations

The following abbreviations are used in this manuscript:

AIDA-I	adhesin involved in diffuse adherence I
AUC	area under the curve
AUC(ELISA)	area under the ELISA dilution curve
AUC(FLOW)	area under the flow cytometry binding curve
bNAbs	broadly neutralizing antibodies
BSA	bovine serum albumin
BSL-2	biosafety level 2
CD4	cluster of differentiation 4
CI	confidence interval
DNA	deoxyribonucleic acid
EC50	half-maximal effective concentration
eHIAs	extended Hia-derived spacer
ELISA	enzyme-linked immunosorbent assay
Env	envelope glycoprotein
FBS	fetal bovine serum
FCS	flow cytometry standard
GCN4	general control nonderepressible 4
gp41	glycoprotein 41
GRB	genome-reduced bacteria
HBSS	Hank’s Balanced Salt Solution
HET3	heterogeneous stock 3
Hia	Haemophilus influenzae adhesin
HIV-1	human immunodeficiency virus type 1
HRP	horseradish peroxidase
IBC	Institutional Biosafety Committee
ID50	50% inhibitory dilution
ID80	80% inhibitory dilution
IgG	immunoglobulin G
KanR	kanamycin resistance gene
KWC/GRB	killed whole-cell genome-reduced bacteria
LB	lysogeny broth
mAb	monoclonal antibody
MPER	membrane-proximal external region
NIAID	National Institute of Allergy and Infectious Diseases
NCI	National Cancer Institute
NIH	National Institutes of Health
OD600	optical density at 600 nm
Ori	origin of replication
PADRE	Pan-DR epitope
PBS	phosphate-buffered saline
PCR	polymerase chain reaction
pLDDT	predicted local distance difference test
PrEP	pre-exposure prophylaxis
pRHIA4	plasmid vector containing the Hia trimeric autotransporter cassette
pTM	predicted template modeling score
p_rhaBAD	rhamnose-inducible promoter
RLUs	relative luminescence units
RRID	Research Resource Identifier
rSIP	recombinant Group B streptococcal surface immunogenic protein
SEM	standard error of the mean
SOC	super optimal broth with catabolite repression
TAE	Tris-acetate-EDTA
TBST	Tris-buffered saline with Tween-20
TLR	Toll-like receptor
TLR2	Toll-like receptor 2
TLR4	Toll-like receptor 4
TMB	3,3′,5,5′-tetramethylbenzidine
TZM-bl	HeLa-derived HIV reporter cell line
USDA	United States Department of Agriculture
NIFA	National Institute of Food and Agriculture
UV	ultraviolet
UVA	University of Virginia
