# Supplementary material for: Trimeric Class I Viral Fusion Protein Vaccine Immunogens Using a Trimeric Autotransporter in a Killed Whole-Cell Bacteria Vaccine Platform: Applications to HIV MPER
Source: Viruses. 2026 Aug 13;18(8):890. doi: 10.3390/v18080890 (PMC13517434; doi:10.3390/v18080890)
Supplement: Supplementary file 1 [file viruses-18-00890-s001.zip › Supplementary Materials/Table_S1_AlphaFold_confidence.pdf]

Table S1. AlphaFold2-Multimer model confidence metrics for all vaccine designs.

Design (N→C)	Stage	Per-domain confidence (pLDDT tier)	pTM	ipTM
M	0	Confident (70–90): Hia. Low (50–70): MPER.	0.62	0.65
M-F	1	Confident (70–90): Hia, Foldon. Low (50–70): MPER.	0.58	0.6
M-Z	1	Confident (70–90): Hia, Zipper. Low (50–70): MPER.	0.54	0.57
M-F-r	2	Confident (70–90): Hia, Foldon, rSIP (structured core). Low (50–70): MPER, rSIP (flexible regions).	0.38	0.4
M-Z-r	2	Confident (70–90): Hia, Zipper, rSIP (structured core). Low (50–70): MPER, rSIP (flexible regions).	0.39	0.42
M-F-r-P	3	Confident (70–90): Hia, Foldon, rSIP (structured core). Low (50–70): MPER, PADRE, rSIP (flexible regions).	0.38	0.39
M-Z-r-P	3	Confident (70–90): Hia, Zipper, rSIP (structured core). Low (50–70): MPER, PADRE, rSIP (flexible regions).	0.37	0.38
M-M-F-r-P	4	Confident (70–90): Hia, Foldon, rSIP (structured core). Low (50–70): MPER (×2), PADRE, rSIP (flexible regions).	0.33	0.34
M-M-Z-r-P	4	Confident (70–90): Hia, Zipper, rSIP (structured core). Low (50–70): MPER (×2), PADRE, rSIP (flexible regions).	0.37	0.38
M-F-M-F-r-P	5	Confident (70–90): Hia, Foldon (×2), rSIP (structured core). Low (50–70): MPER (×2), PADRE, rSIP (flexible regions).	0.30	0.31
M-F-M-F-r-P eHIAs	6	Confident (70–90): Hia, Foldon (×2), rSIP (structured core). Low (50–70): MPER (×2), PADRE, eHIAs, rSIP (flexible regions).	0.34	0.33

Per-residue confidence (pLDDT) is summarized by structural domain using standard AlphaFold confidence tiers (very high, pLDDT > 90; confident, 70–90; low, 50–70; very low, < 50). Global (pTM) and interface (ipTM) confidence scores correspond to the top-ranked model of each design. Domain composition for each construct is given in Table 2 of the main text. Across all designs, individual folded scaffold domains (the Hia β-barrel and the Foldon/Zipper trimerization domains) were modeled in the confident range, whereas the functionally critical MPER antigen and the flexible spacer and immunomodulatory elements (eHIAs, PADRE, and portions of rSIP) fell in the low-confidence range. Global and interface confidence were consistently low across all constructs (pTM ≈ 0.33–0.34; ipTM ≈ 0.32–0.34), indicating that although local domain folds were predicted with reasonable confidence, the overall multidomain and inter-chain arrangement was not confidently predicted—as expected for large, elongated, membrane-associated trimeric assemblies containing intrinsically flexible segments. Structural models were therefore used qualitatively, as hypothesis-generating design rationale, rather than as structural evidence;

all functional conclusions rest on the experimental flow cytometry, ELISA, and pseudovirus neutralization data.
